# Microoxic conditions promote *Escherichia*-associated cellulase expression in the giant panda gut

**DOI:** 10.1093/ismejo/wrag068

**Published:** 2026-04-02

**Authors:** Feilong Deng, Yanhua Han, Yunjuan Peng, Zhijian Xu, Jianbo Yang, Jinling He, Desheng Li, Guixin Dong, Peng Zhang, Hui Jiang, Jianmin Chai, Chengdong Wang, Jiangchao Zhao, Ying Li

**Affiliations:** Guangdong Provincial Key Laboratory of Animal Molecular Design and Precise Breeding, School of Animal Science and Technology, Foshan University, Foshan 528225, China; Guangdong Provincial Key Laboratory of Animal Molecular Design and Precise Breeding, School of Animal Science and Technology, Foshan University, Foshan 528225, China; College of Animal Science, South China Agricultural University, Guangzhou 510642, China; Guangdong Provincial Key Laboratory of Animal Molecular Design and Precise Breeding, School of Animal Science and Technology, Foshan University, Foshan 528225, China; Guangdong Provincial Key Laboratory of Animal Molecular Design and Precise Breeding, School of Animal Science and Technology, Foshan University, Foshan 528225, China; Guangdong Provincial Key Laboratory of Animal Molecular Design and Precise Breeding, School of Animal Science and Technology, Foshan University, Foshan 528225, China; China Conservation and Research Center for the Giant Panda, Key Laboratory of SFGA on the Giant Panda, Chengdu 611830, China; Southern China Wildlife Species Conservation Center, Zhuhai 519031, China; Southern China Wildlife Species Conservation Center, Zhuhai 519031, China; Guangdong Provincial Key Laboratory of Animal Molecular Design and Precise Breeding, School of Animal Science and Technology, Foshan University, Foshan 528225, China; Guangdong Provincial Key Laboratory of Animal Molecular Design and Precise Breeding, School of Animal Science and Technology, Foshan University, Foshan 528225, China; China Conservation and Research Center for the Giant Panda, Key Laboratory of SFGA on the Giant Panda, Chengdu 611830, China; College of Animal Science, South China Agricultural University, Guangzhou 510642, China; Guangdong Provincial Key Laboratory of Animal Molecular Design and Precise Breeding, School of Animal Science and Technology, Foshan University, Foshan 528225, China

**Keywords:** microbial scRNA-seq, *Escherichia coli*, host-microbe coadaptation, giant panda gut microbiota

## Abstract

Giant pandas possess a carnivore-like gastrointestinal tract yet subsist on bamboo, and their gut communities contain few canonical cellulolytic taxa. We investigated how fiber processing proceeds in this setting by building a species-resolved reference and linking community features to cellular transcriptional profiles and isolate phenotypes. Using culturomics and PacBio HiFi metagenomics, we assembled a species-resolved reference catalog for the panda gut microbiome (Pbac v2; 466 species-level genomes). Community profiling across 142 samples resolved three enterotypes dominated by *Escherichia coli* (ET-Ecoli), *Clostridium* SGBP116 (ET-Clos), and *Streptococcus alactolyticus* (ET-StreA), with ET-Ecoli enriched for tricarboxylic-acid and respiratory-chain modules and showing higher abundance of an endo-β-1,4-glucanase marker. Droplet-based microbial single-cell RNA-seq from four samples (16 659 cells) assigned a substantial share of cellulase-associated transcripts (GH1/GH3/GH5/GH9) *in situ* to *Escherichia* and revealed within-species heterogeneity: *E. coli* subpopulations segregated into respiration-enriched versus three-carbon/anaerobic-like programs, with cellulase/lytic polysaccharide monooxygenase-linked transcripts concentrated in the former. Guided by these associations, panda-derived *E. coli* isolates assayed under defined atmospheres showed oxygen-dependent cellulolytic readouts *in vitro*. Although *in vivo* oxygen levels were not measured, the convergence of species-resolved community signatures, single-cell attribution, and isolate phenotypes indicates that *E. coli* can contribute to cellulose processing under microoxic conditions in this cohort. The Pbac v2 resource and the integrated workflow (culturomics + HiFi metagenomes, multi-omics, microbial scRNA-seq) provide a template for species-level assignment of microbiome functions in hosts with unconventional diet–physiology combinations.

## Introduction

Giant pandas (*Ailuropoda melanoleuca*), phylogenetically within Ursidae, retain a carnivore-adapted gastrointestinal tract with a short intestine and rapid transit [1, 2]. Paradoxically, their diet consists almost exclusively of bamboo, a fiber-rich, nutrient-poor resource dominated by cellulose and hemicellulose, whereas the panda genome encodes no endogenous cellulose/hemicellulose hydrolases [3, 4]. This presents a central question in host-microbe adaptation: which microbial taxa and physiological states are involved in bamboo fiber processing in a gastrointestinal tract with a carnivore-like architecture, and under which ecological conditions are these activities likely to occur? Early studies implicated the gut microbiota in processing bamboo polysaccharides, reporting cellulase-related activities or gene content in panda samples [5–8]. In contrast, community surveys consistently indicated that panda gut microbiomes resemble those of carnivores more than herbivores and are depleted of canonical fibrolytics [9, 10]; our prior integrated metagenomic/meta-transcriptomic analyses likewise suggested limited cellulose/hemicellulose capacity and a functional profile closer to carnivores [11]. Despite rapid transit [2], a fraction of fiber is nonetheless degraded, leaving unresolved which organisms perform fiber-associated functions, under what ecological conditions they do so, and whether within-species functional heterogeneity contributes. Indeed, digestion trials indicate that giant pandas degrade only a limited fraction of bamboo, with bamboo dry-matter digestibility averaging <20% and apparent digestion coefficients of ~27% for hemicellulose and ~8% for cellulose [2]. Moreover, putative cellulolytic isolates from the panda gastrointestinal tract have lacked *in situ* functional attribution [5, 9, 12].

In this study, we address these gaps with a species-resolved framework that links community structure to cellular states and isolate-level function. First, we integrated culturomics with PacBio HiFi metagenomics to assemble an enhanced genomic reference for the panda gut (Pbac v2; 466 species-level genomes). Second, using this reference, we delineated species-level enterotypes and associated carbon/energy modules. Third, informed by anatomical and ecological context that may permit transient microoxic niches, we examined whether oxygen availability modulates cellulase-associated phenotypes in panda-derived *Escherichia* isolates *in vitro*, and we applied microbial single-cell RNA-seq to assign cellulase-associated transcripts *in situ*. These approaches reveal functional heterogeneity within *Escherichia coli*, with coexisting respiration-linked and three-carbon/anaerobic-like states in which cellulase/lytic polysaccharide monooxygenase (LPMO) programs track the former, and provide evidence that under microoxic conditions in this cohort, *E. coli* can contribute to cellulose breakdown. The resulting genomic and single-cell resources support species-level analyses of functional adaptation in this specialized symbiosis.

## Materials and methods

### Sample collection of giant pandas

Fecal samples were collected from captive giant pandas housed at the Chengdu Research Base of Giant Panda Breeding. Ten fecal samples (one per individual) were used for PacBio HiFi metagenomic sequencing (Supplementary Table S1). Four additional fecal samples were collected for microbial single-cell RNA sequencing (scRNA-seq) and were also profiled using multiple sequencing approaches, including PacBio HiFi metagenomic sequencing, Illumina short-read metagenomic sequencing, and 16S rRNA gene sequencing (Supplementary Table S1). Moreover, three PacBio HiFi metagenomic datasets generated in our previous study (accession number: PRJNA1073587) were included, resulting in a total of 17 HiFi metagenomic datasets in this study (Supplementary Table S1).

Fresh fecal samples from healthy giant pandas and postmortem gastrointestinal contents from two deceased giant pandas were used for cultivation-based microbial isolation/culturomics (Supplementary Table S2). For the deceased individuals, intestinal contents were collected from the stomach, duodenum, jejunum, ileum, colon, and rectum.

### DNA extraction, library preparation, and metagenomic sequencing

Total DNA was extracted from panda feces that were snap-frozen in liquid nitrogen immediately after collection and stored frozen until processing; samples were thawed on ice prior to extraction using the Magnetic Universal Genomic DNA Kit (QIAGEN, Beijing). Quality was assessed by 1% agarose gel and concentration by Qubit 3.0. Samples with a primary band >30 kb were sheared by Covaris to 15–18 kb, size-enriched with magnetic beads, and subjected to damage repair and end-repair. Stem-loop (SMRTbell) adapters were ligated; unligated DNA was removed by exonucleases. Libraries were sequenced on the PacBio Revio platform (Novogene). For short reads, libraries were prepared with the NEBNext Ultra DNA Library Prep Kit for Illumina and sequenced on a NovaSeq 6000 System (Illumina), 150 bp paired-end.

### Reconstruction of metagenomic assembled genomes by different assembly methods

The KneadData pipeline v0.7.2 (https://bitbucket.org/biobakery/kneaddata) was used for quality control (QC) and host removal of Illumina short reads. Within KneadData, Trimmomatic v0.39 [13] discarded low-quality reads, and Bowtie2 filtered host (GCF_002007445.1) and diet contaminants (GCA_017311315.1; GCA_011038535.1) [14]. Filtered reads were assembled with metaSPAdes (--meta, --only-assembler) [15].

For PacBio HiFi data, polymerase reads were converted to subreads on the Revio platform, then then circular consensus sequence (CCS) reads were generated with SMRT Link (--min-passes 3; --min-rq 0.99). HiFi reads were screened against host (GCF_002007445.1) and diet references (GCA_017311315.1; GCA_011038535.1) using minimap2 (map-pb) [16]. PacBio HiFi Long-read assemblies were generated using Flye v2.9.2 with --pacbio-hifi option. Previously published ONT (Oxford Nanopore Technologies) long-read datasets were downloaded from BioProject PRJNA1073587 [17] and reassembled using Flye v2.9.2 with the --nano-raw option.

Contigs were binned with MetaBAT2 v2.12.1 (default) [18]. Bins >500 kb were retained. Redundant bins within each assembly strategy were dereplicated with dRep v3.4.3 (−sa 0.99) [19]. Completeness and contamination were assessed with CheckM2 [20]; only medium/high-quality metagenome-assembled genomes (MAGs) (completeness >50%, contamination <10%) were kept. rRNA and tRNA genes were annotated with Barrnap (https://github.com/tseemann/barrnap) and tRNAscan-SE [21], respectively. Taxonomy was assigned with GTDB-Tk v2.1.0 [22] using GTDB release R220 (accessed 18 September 2024).

Species phylogenies were inferred from MAGs using PhyloPhlAn 3.0 (v3.0.2), which builds trees from core-gene SNPs [23], and visualized in iTOL v6.5.2 [24].

### Isolation, whole genome sequencing, and assembly

Microbial isolation and cultivation were performed using fecal samples from healthy giant pandas and postmortem gastrointestinal contents collected from two deceased individuals (a captive individual that died unexpectedly during anesthesia and a wild individual that died accidentally; Supplementary Table S2). A variety of culture media and incubation conditions (Supplementary Table S3) were employed to maximize the diversity of cultivable microorganisms from the giant panda intestinal tract. All isolates were verified as clonal by repeated single-colony streaking (including re-streaking from frozen stocks prior to sequencing), and only well-isolated colonies with uniform morphology were selected for expansion.

Pure isolates were obtained by repeated single-colony streaking. For each isolate, genomic DNA was extracted from pure cultures using a DNA extraction kit (KG203, TIANGEN BIOTECH Co., Ltd, Shanghai, China) following standard bacterial DNA extraction procedures according to the manufacturer’s protocol. DNA quality and quantity were assessed prior to library construction. Sequencing libraries were prepared and genomic DNA was sequenced on a NovaSeq 6000 System (Illumina), targeting a minimum depth of 100× coverage per genome. After reads quality filtering, draft genomes were de novo assembled from the Illumina reads using SPAdes with default parameters.

### Taxonomic, functional profiling, and enterotyping

To quantify bacterial abundances, we constructed a custom Kraken 2-compatible database using the 466 representative genomes [25]. Taxonomic classification for both metagenomic and meta-transcriptomic reads was performed with Kraken 2 (v2.1.2), and species-level abundance estimation refined with Bracken (v2.9) [26]. A total of 142 metagenomic datasets generated via Illumina short-read metagenomic sequencing (Supplementary Table S4) were included for species-level relative abundance profiling. Enterotypes were classified using Partitioning Around Medoids (PAM) clustering based on Bray–Curtis dissimilarity of species-level abundances derived from metagenomic profiles. To evaluate the stability and generalizability of the enterotype classification, we assessed clustering performance across a range of cluster numbers (k = 2–6). Species-level relative abundance data were Hellinger-transformed using the vegan R package, and clustering quality was evaluated using two complementary indices: the Calinski–Harabasz index, calculated from k-means clustering on the transformed data (Euclidean space), and the mean silhouette width, derived from PAM clustering based on Bray–Curtis dissimilarities. Both indices were computed iteratively across k values using 100 random initializations to ensure robustness. To further assess cluster reproducibility, bootstrap resampling was performed (B = 500) with the clusterboot() function in the fpc package.

Gene prediction was performed on the 466 genomes using Prodigal v2.6.3. A non-redundant pangenome catalog was generated by clustering predicted genes with CD-HIT-EST v4.8.1 at 95% nucleotide identity threshold [27]. Functional annotation was implemented via eggNOG-mapper v2.1.12 using default parameters [28]. Gene expression quantification was conducted with Salmon v1.9.0 in mapping-based mode [29]. Differential functional gene clusters across enterotypes were identified using LEfSe (Linear Discriminant Analysis Effect Size) with significance thresholds of *P* < .05 and LDA score >3.0.

### Identification of candidate cellulose-degrading microbes

A multi-step bioinformatics pipeline was implemented to screen cellulose-degrading microorganisms from giant panda gut metagenomes. Genes of each genome were predicted using prodigal software with default parameters. Protein-coding sequences were annotated for carbohydrate-active enzymes using dbCAN3 [30] (E-value <1e-5, coverage ≥70%) with reference to the CAZy database [31]. Glycoside hydrolase (GH) families were systematically screened, including cellulose-targeting enzymes (GH5, GH9, GH48, GH6, GH7, GH1, GH3, GH8, GH16).

Genomes were classified based on enzymatic machinery completeness. For cellulose degradation, candidates were required to encode: (i) ≥1 endoglucanase (GH5/GH8), (ii) ≥1 exo-acting/processive cellulase (GH48/GH6/GH7/GH9/GH16), and (iii) ≥1 β-glucosidase (GH1/GH3). A quantitative scoring system was established to evaluate the functional potential of microbial genomes for polysaccharide degradation. Because complete cellulose hydrolysis is generally achieved through the concerted action of endoglucanases, exoglucanases/cellobiohydrolases, and β-glucosidases [32], we used the joint presence of these three functional components as a conservative operational criterion for candidate cellulose-degrading genomes. Genomes meeting this criterion were assigned a baseline score of 1 point. Additional points were allocated based on three categorical criteria: (a) presence of LPMOs (AA9/AA10 families), (b) functional redundancy in endoglucanase genes (≥2 copies), and (c) functional redundancy in exoglucanase genes (≥2 copies), with each criterion contributing one point (maximum three points). LPMO-like hits and gene-copy redundancy were treated as confidence-enhancing features rather than core requirements.

### Microbial single-cell RNA-seq

#### Cell suspension

Fresh panda stool was fixed in 4% paraformaldehyde (PFA), vortexed, sequentially filtered (70 μm, then 10 μm strainers), centrifuged (4000 × g, 10 min, 4°C), resuspended in 4% PFA, and incubated overnight (4°C, shaking).

#### Permeabilization

Fixed cells were washed in phosphate-buffered saline (PBS) with RNase inhibitor (1 U/μl) and 100 mM Tris–HCl (pH 7), permeabilized with 0.04% Tween-20 on ice (3 min), then digested with lysozyme (VITAPilote-PFT1200; 147.5 μl cells, 40 μl Lyso-Buffer, 2.5 μl RNase inhibitor, 10 μl lysozyme; 37°C, 15 min). Reactions were stopped with PBS-RI and 5 × 10^6^ cells collected.

#### *In situ* pre-barcoding reverse transcription

Aliquots of cells were reverse-transcribed with pre-barcoded random primers (PFT1200), deoxynucleoside triphosphates (dNTPs), and reverse transcriptase; 12 annealing cycles (8–42°C) and a 42°C, 30-min extension were followed by ethylenediaminetetraacetic acid (EDTA) (50 mM). cDNA received dA-tailing with TT enzyme and dATP (100 mM) at 37°C for 30 min.

#### Droplet generation

Cells, hydrogel barcoded beads, and extension mix were co-encapsulated on a VITAcruizer DP400; incubation was 37°C (1 h), 50°C (30 min), 60°C (30 min), 75°C (20 min).

#### cDNA purification/amplification

Droplets were broken with perfluorooctane; cDNA was purified (AMPure XP), polymerase chain reaction (PCR)-amplified, and quantified (Qubit 4.0; Agilent 4200).

#### Library prep/sequencing

Libraries (VAHTS Universal DNA) underwent end-repair, A-tailing, and adapter ligation, and were sequenced on a NovaSeq 6000 System (Illumina), 150 bp paired-end.

### Data analysis for microbial scRNA-seq

#### Single microbe annotation

Reference genomes were drawn from the Pbac v2 catalog generated in this study, retaining entries with >80% completeness and <5% contamination. For each barcode, reads were classified with Kraken 2 (v2.1.2) [25] against this reference and refined to species-level abundances with Bracken [26]. The species with the highest relative abundance was assigned as the barcode’s identity; this value defined barcode purity. Barcodes with purity ≥50% were kept for downstream analyses.

#### Single microbe bacteria analysis

Raw paired-end sequencing data underwent primer and dA-tailed base trimming. Reads were aligned to the Pbac v2 genome database using STARsolo in STAR (v2.7.10a) [33] with standardized parameters. To avoid ambiguity across orthologous loci, only high-quality uniquely mapped reads were retained for unique molecular identifier (UMI) counting, and multi-mapping reads were discarded. Gene identifiers in the reference carried genome-level prefixes (GenomeID|GeneID), so that all gene entries in the expression matrix remained species-specific. Gene expression matrices were generated from UMI counts. Barcodes with nFeature_RNA <15 were filtered out prior to downstream analysis. Data processing utilized Seurat 4.3.0 [34]. The marker genes with a log2 fold change >0.25 and an adjusted *P*-value <.05 were considered significantly differentially expressed genes. Species identities were assigned at the cell (barcode) level using the Kraken 2/Bracken-based majority assignment described above, and gene-level UMI counts were then quantified per barcode, such that taxonomic annotation and expression quantification are conceptually decoupled.

#### Oxygen-dependence scoring (O₂ score)

To quantify single-cell dependence on oxidative metabolism, we constructed a bidirectional gene-set score contrasting markers of aerobic respiration with markers of microoxic/anaerobic respiration and fermentation. The aerobic set (O₂^+^) comprised *cyoA/B/C/D*, *sdhA/B/C/D*, and core tricarboxylic acid (TCA) cycle genes (icd, *sucA/B/C/D*). The anaerobic/fermentative set (O₂^−^) comprised *cydA/B/X*, *frdA/B/C/D*, *narG/H/I/J* and *nrfA*, and fermentation-associated genes (*pflB*, *ldhA*, *adhE*). Features were mapped to genes via the EggNOG annotation. To ensure stability, each set was required to match at least three features. Using Seurat (RNA assay; log-normalized expression), we computed module scores for O₂^+^ and O₂^−^ using AddModuleScore with default parameters in Seurat (slot = “data,” nbin = 24, ctrl = 100, k = FALSE, seed = 1, default feature pool) and defined O₂ score = score(O₂^+^) − score(O₂^−^). For comparability and visualization, O₂ score values were z-standardized to yield O₂ score_z.

#### Trajectory inference and lineage definition

Analyses were restricted to *E. coli* cells. The RNA assay was normalized and log-transformed, highly variable features were selected, principal component analysis (PCA) was performed, and a nearest-neighbor graph and uniform manifold approximation and projection (UMAP) embedding were computed. Clusters were taken from seurat_clusters, and samples for grouping/random effects were identified by orig.ident. To reduce single-sample idiosyncrasies, only clusters present in ≥2 samples were retained; seurat_clusters was treated as a factor for stratified visualization and mixed-effects modeling. Trajectory inference used slingshot with UMAP coordinates as input. A minimum spanning tree was constructed on cluster centers, and principal curves were fitted to obtain lineage assignments (lineage_assign) and continuous pseudotime (slingPseudotime). The tradeSeq package was applied to model gene expression along pseudotime for heatmaps and representative smoothed curves; cells were ordered by pseudotime of the target lineage for plotting.

#### Cell-level correlation analysis

At the single-cell level, Spearman correlations were computed between O_2_ score_z and enzyme/signature scores (Endo, Exo, Beta_glu, LPMOs), using pairwise-complete observations. Multiple testing was controlled by the Benjamini–Hochberg false discovery rate (FDR). Correlation matrices were visualized with ComplexHeatmap; distances were defined as 1−ρ, symmetrized and truncated to non-negative values before hierarchical clustering, and cells with FDR <0.05 were marked with asterisks.

### Functional validation of cellulolytic activity

Frozen strains were revived in Luria–Bertani (LB) (30 ml, 37°C). Aliquots (2–5 μl) were spotted on 1% sodium carboxymethyl cellulose (CMC-Na) agar and incubated 24 h at 37°C. Plates were stained with Congo red (1 mg/ml, 30 min), destained with 1 M NaCl (30 min), and halos recorded (triplicates). For activity, a glucose standard (0.125–2 mg/ml) was mixed 1:1 with 3,5-dinitrosalicylic acid (DNS) reagent, boiled for 15 min, and the absorbance was measured at 540 nm. Crude enzyme solution was prepared by collecting the supernatant after bacterial growth in LB medium (12 h), followed by transfer to fermentation medium for 24 h at 37°C and centrifugation at 8000 × g for 20 min. Reactions containing 1 ml crude enzyme solution and 1 ml 1% CMC-Na were incubated at 37 °C for 30 min, mixed with 2 ml DNS, boiled for 15 min, diluted to 10 ml, and the absorbance at 540 nm was measured against enzyme-free controls; enzyme activity was calculated from the standard curve. CMC-Na depletion was assessed by quantifying residual polymer in culture supernatants. Six *E. coli* strains were cultivated under 0%, 4%, or 21% O₂ using a Don Whitley Scientific M35 workstation and inoculated into medium containing 1% (w/v) CMC-Na for 24 h. Cultures were centrifuged (8800 × g, 10 min, 4°C), and supernatants were filtered (0.22 μm). One milliliter of filtrate was mixed with three volumes of pre-chilled absolute ethanol (final ~75% v/v) and incubated at 4°C overnight to precipitate CMC-Na. Precipitates were collected by centrifugation (8800 × g, 10 min, 4°C), washed once with 70% ethanol and once with absolute ethanol, lyophilized to constant weight, and weighed (m₁). Background precipitate from CMC-free medium processed in parallel was subtracted (m = m₁ − m₀), and values were corrected for recovery using CMC-spiked controls.

### Transcriptomics for *E. coli*

Gene expression profiles of six giant panda-derived *E. coli* strains were compared under 0% versus 4% O₂ conditions. Strains were cultured in CMC-Na medium under the indicated oxygen conditions and harvested by high-speed centrifugation after 24 h (early stationary phase) of cultivation, corresponding to the time point used for the cellulose-processing assays.

#### Transcriptomics

RNA was extracted using RNAprep Pure Cell/Bacteria Kit. RNA integrity was verified via 1% agarose gel electrophoresis and Bioanalyzer 2100 assessment. Ribosomal RNA was depleted from total RNA, followed by cDNA synthesis using random hexamers with deoxyuridine triphosphate (dUTP) incorporation for strand specificity. Libraries were prepared through end repair, A-tailing, adapter ligation, size selection, USER enzyme digestion, and PCR amplification. Quantification utilized Qubit fluorometry and real-time PCR, with size distribution verified by bioanalyzer. Indexed libraries were clustered on a cBot Cluster Generation System (TruSeq PE Cluster Kit v3) and sequenced on a NovaSeq 6000 System (Illumina).

#### Bioinformatics for RNA-seq

Gene prediction for all six strains was performed with Prokka v1.14.6 [35]. A pangenome reference was constructed using Roary v3.11.2 [36]. RNA-seq reads underwent quality control via Fastp v0.19.5 [37], alignment to the pangenome using HISAT2 v2.1.0 [38], and gene quantification via SAMtools v1.17 [39] and featureCounts [40].

## Results

### Integrated expansion of cultured isolates and HiFi-assembled genomes builds a species-resolved reference

**Figure 1:**
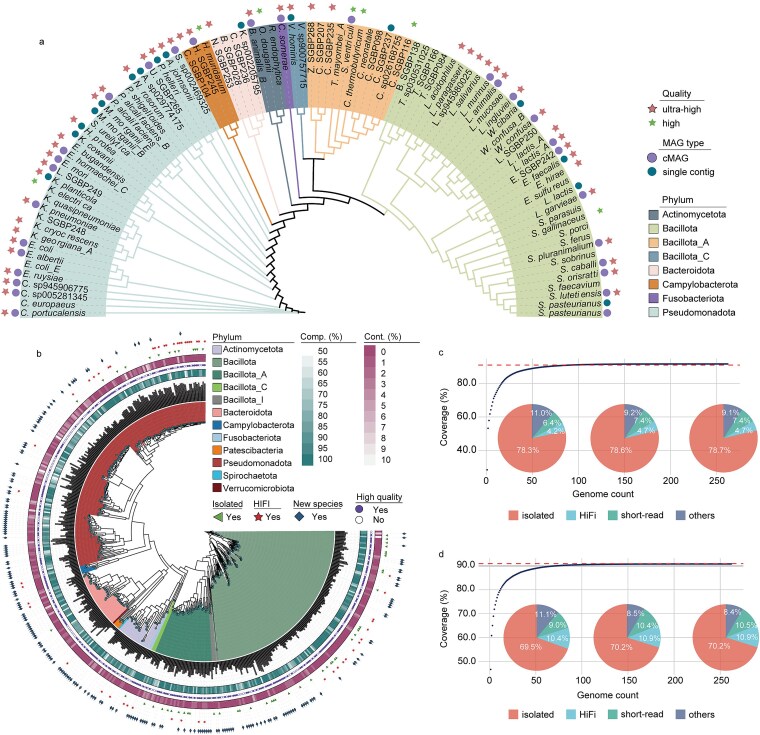
**Construction of a non-redundant, species-resolved reference (Pbac v2) for the giant panda gut microbiome using culturomics and HiFi metagenomic sequencing;** (a) MAGs assembled using HiFi long reads from all 17 PacBio HiFi libraries; MAGs were categorized as cMAGs (circularized single-contig), single-contig genomes(non-circular), and multi-contig MAGs (≥2 contigs); MAGs with completeness greater than 99% and contamination below 3% were classified as ultra-high-quality MAGs; (b) phylogenomic tree of the final set of 466 non-redundant species-level genomes, generated by clustering and dereplicating all genomes (PacBio HiFi MAGs, short-read-derived MAGs, and isolate genomes) at 95% average nucleotide identity (ANI); of the 466 genomes, 62 are isolate genomes and 44 are PacBio HiFi MAGs generated in this study; concentric rings from inner to outer indicate: (1) genome size; (2) completeness (Comp., CheckM2); (3) high-quality (marked by circles, with ≥90% completeness and <5% contamination); (4) contamination level (Cont., purple gradient); (5) culture status (isolated strains); (6) assembly source (PacBio HiFi-derived genomes); (7) novelty (ANI <95% relative to existing databases; *n* = 213); (c) metagenomic coverage dynamics with expanding genome subsets; the horizontal dashed line marks the reference value of 0.91; x-axis: top *n* most abundant genomes; y-axis: cumulative alignment rate; inset pie charts (*n* = 50/150/250) show source composition; (d) meta-transcriptomic coverage dynamics with expanding genome subsets; the red dashed line marks the reference line at 0.91; x-axis: top *n* most abundant genomes; y-axis: cumulative alignment rate; inset pie charts (*n* = 50/150/250) show source composition; percentages in the pie charts indicate the fraction of genomes (by genome count) within the top *n* most abundant genomes that originate from each source category (isolated, HiFi, short-read, and others).

Across 22 giant panda gastrointestinal samples (9 fecal samples from healthy individuals and 13 gastrointestinal content samples collected from the stomach, duodenum, jejunum, ileum, colon, and rectum of two deceased pandas; Supplementary Table S2), large-scale culturomics yielded 9041 colonies. Following identification by matrix-assisted laser desorption/ionization time-of-flight (MALDI-TOF) and 16S rRNA gene Sanger sequencing, de-replication based on 16S rRNA gene sequence data yielded 99 isolates for genome sequencing. These represent 76 nonredundant strains [≥99% average nucleotide identity (ANI)], spanning 62 species (≥95% ANI), 39 genera, 18 families, and 6 phyla, including three previously unclassified species-level clusters (one *Limosilactobacillus* and two *Staphylococcus*) (Fig. 1a; Supplementary Table S5). These 99 genomes are publicly accessible and provide isolate anchors for downstream functional analyses (Supplementary Fig. S1).

To complement isolate collection and broaden community-level coverage, we generated 17 PacBio HiFi metagenomic datasets (post-QC: 44.45 million long reads; 449.11 Gb; N50 10 734 bp; Supplementary Table S1). Using the long and accurate HiFi reads, assembly and binning yielded high-contiguity, high-completeness reconstructions: 183 nonredundant MAGs (99% similarity) across 93 species clusters, including 69 near-complete MAGs (≥99% completeness, <3% contamination) and 49 single-contig genomes (Fig. 1a; Supplementary Table S6), supporting their use as species-resolved references in this cohort.

Integrating isolate genomes with HiFi-assembled MAGs, we updated Pbac v1 to a dereplicated, species-resolved reference catalog (Pbac v2, Fig. 1b). All genomes from PacBio HiFi MAGs, Illumina short-read MAGs, and isolate genomes were clustered at 95% ANI and dereplicated across sequencing platforms, yielding 466 non-redundant species-level genomes spanning 187 genera, 74 families, and 12 phyla (Supplementary Table S7, Supplementary Fig. S2a). Among these, 213 species-level representatives and 5 genera lacked ≥95% ANI matches to current databases (Fig. 1b). When used as a reference, high-quality genomes (≥90% completeness, <5% contamination) aligned on average 92.7% of metagenomic and 91.9% of meta-transcriptomic reads (*n* = 14 samples; Supplementary Fig. S2b); relaxing completeness to ≥80% increased coverage to 96.5% (metagenomic, MG) and 96.2% (meta-transcriptomic, MT). The top 50 genomes captured 88.9% (MG) and 89.0% (MT) of reads (Fig. 1c and d), with 82.5% and 79.9% of those top genomes, respectively, originating from isolates or HiFi assemblies, supporting efficient, species-resolved profiling in this cohort.

### Functional specialization and core metabolism across enterotypes

**Figure 2:**
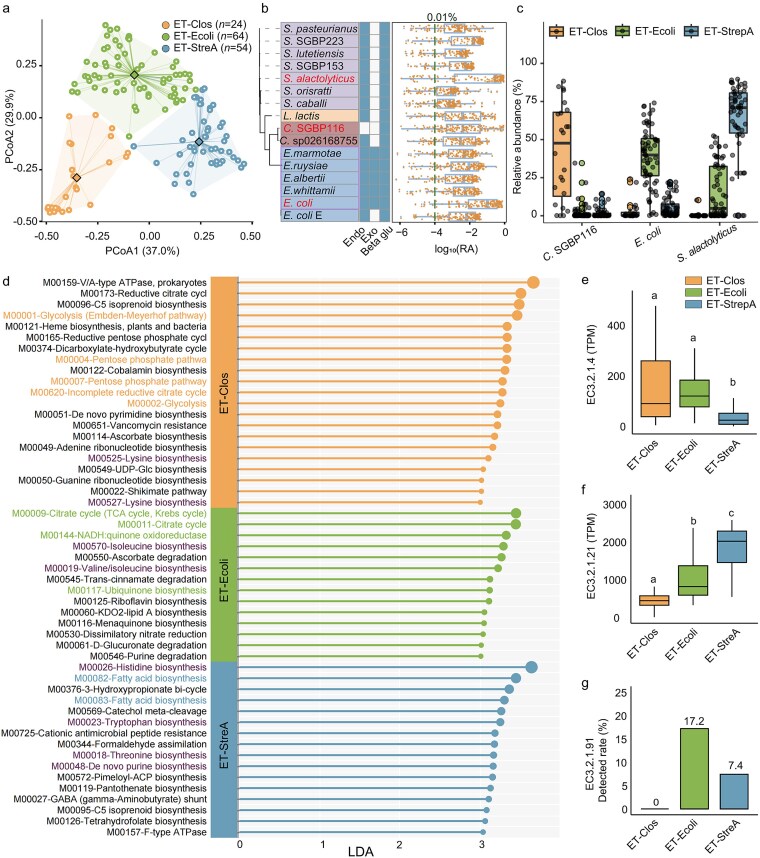
**Core species, community structure, and functional modules of enterotypes**; (a) PCoA plot based on Bray–Curtis dissimilarity of three enterotypes: ET-Clos (orange circles; dominated by *Clostridium* SGBP116), ​ET-Ecoli (green circles; dominated by *Escherichia coli*), and ET-StreA (blue circles; dominated by *Streptococcus alactolyticus*); (b) phylogenetic tree of core gut bacteria (defined as taxa present in >70% of samples with relative abundance >0.01%) and their relative abundance across 142 samples; background colors represent bacterial genera; red labels highlight dominant species in each enterotype; heatmap illustrates genomic presence (blue) or absence (white) of endo-cellulase (Endo), exo-cellulase (Exo), and β-glucosidase (Beta glu) in corresponding species; co-detection of all three enzyme-encoding genes indicates functional potential for cellulose degradation; relative abundance is displayed as boxplots (blue) with overlaid jittered points (orange; each point represents a sample); the x-axis is log10-transformed, and the green dashed line indicates the 0.01% abundance threshold; (c) taxon abundance distributions across enterotypes (box plots); taxa: *S. alactolyticus*, *E. coli*, and *Clostridium* SGBP116; colors denote enterotypes; (d) differentially enriched KEGG modules among enterotypes identified by LEfSe analysis; the x-axis shows LDA scores; colored module labels indicate representative modules highlighted in the main text as relatively concentrated in each enterotype (orange, ET-Clos; green, ET-Ecoli; blue, ET-StreA); purple labels denote biosynthetic modules, predominantly amino acid biosynthesis-related pathways; (e-g) comparative abundance of enzymes representing key steps in cellulose degradation across giant panda enterotypes: EC 3.2.1.4 (endo-1,4-β-glucanase; endoglucanase; (e) EC 3.2.1.21 (β-glucosidase; cellobiose/β-glucoside hydrolysis; (f) and EC 3.2.1.91 (cellulose 1,4-β-cellobiosidase; putative exo-cellulase/cellobiohydrolase marker; g); group comparisons were conducted using Kruskal–Wallis tests, with Dunn’s post hoc analysis incorporating Benjamini–Hochberg false discovery rate correction (α = 0.05); significant differences (adjusted *P* < .05) are indicated by unique superscript letters; because EC 3.2.1.91 was zero-inflated, panel (g) reflects its distribution across samples, with the highest detection prevalence observed in ET-Ecoli.

Using Pbac v2 as the reference, species-resolved metagenomic profiling (*n* = 142 samples, Supplementary Table S8) identified three enterotypes: a *Clostridium* SGBP116-dominated enterotype (ET-Clos), an *E. coli-*dominated enterotype (ET-Ecoli), and *Streptococcus alactolyticus-*dominated enterotype (ET-StreA) (Fig. 2a). Clustering evaluation confirmed that a three-cluster solution was statistically optimal and highly stable, as supported by Calinski–Harabasz, silhouette, and bootstrap validation analyses (see Supplementary Note 1 and Supplementary Fig. S3). *Clostridium* SGBP116, assembled in this study and validated across PacBio HiFi, ONT, and Illumina datasets (>99% completeness,<0.5% contamination), represents a previously uncharacterized species (Supplementary Fig. S4; Supplementary Table S6). Applying prevalence (>70%) and abundance (>0.01%) thresholds, *S. alactolyticus* (mean relative abundance across all 142 samples: 30.8%), *E. coli* (19.9%), and *Clostridium* SGBP116 (8.6%) were classified as core taxa (Fig. 2b). Within enterotypes, median relative abundance reached 62.6% in ET-StreA, 38.4% in ET-Ecoli, and 42.2% in ET-Clos (Fig. 2c).

Differential module enrichment identified by LEfSe (Fig. 2d; Supplementary Tables S9 and 10) indicated distinct metabolic emphases. ET-Clos was enriched for glycolysis (M00001), three-carbon metabolism (M00002), and reductive TCA-associated modules (M00173, M00620). ET-Ecoli showed enrichment for TCA modules (M00009, M00011) and electron-transport components, including NADH:quinone oxidoreductase (M00144) and ubiquinone biosynthesis (M00117). ET-StreA exhibited relatively higher abundance of amino acid (M00018, M00023, M00026) and fatty-acid biosynthesis modules (M00082, M00083).

Cellulose-associated enzymes differed across enterotypes (Fig. 2e–g). ET-Clos showed higher relative abundance of EC 3.2.1.4 (putative endo-1,4-β-glucanase; Fig. 2e) than ET-StreA (Benjamini–Hochberg–adjusted *P* < .05). ET-StreA showed higher EC 3.2.1.21 (β-glucosidase; Fig. 2f), consistent with increased capacity for downstream hydrolysis of soluble β-glucosides/cellobiose. EC 3.2.1.91 was sparsely detected overall, but its detection prevalence was highest in ET-Ecoli (Fig. 2g). EC 3.2.1.4 pattern remained consistent under alternative enterotype definitions (Supplementary Note 2). The concurrence of elevated EC 3.2.1.4 with enrichment of TCA and respiratory-chain modules in ET-Ecoli (M00009, M00011, M00144, M00117; Fig. 2d) prompted evaluation of cellulase-associated phenotypes in panda-derived *Escherichia* under controlled conditions.

### *In situ* attribution of cellulase-associated transcripts by microbial scRNA-seq

**Figure 3:**
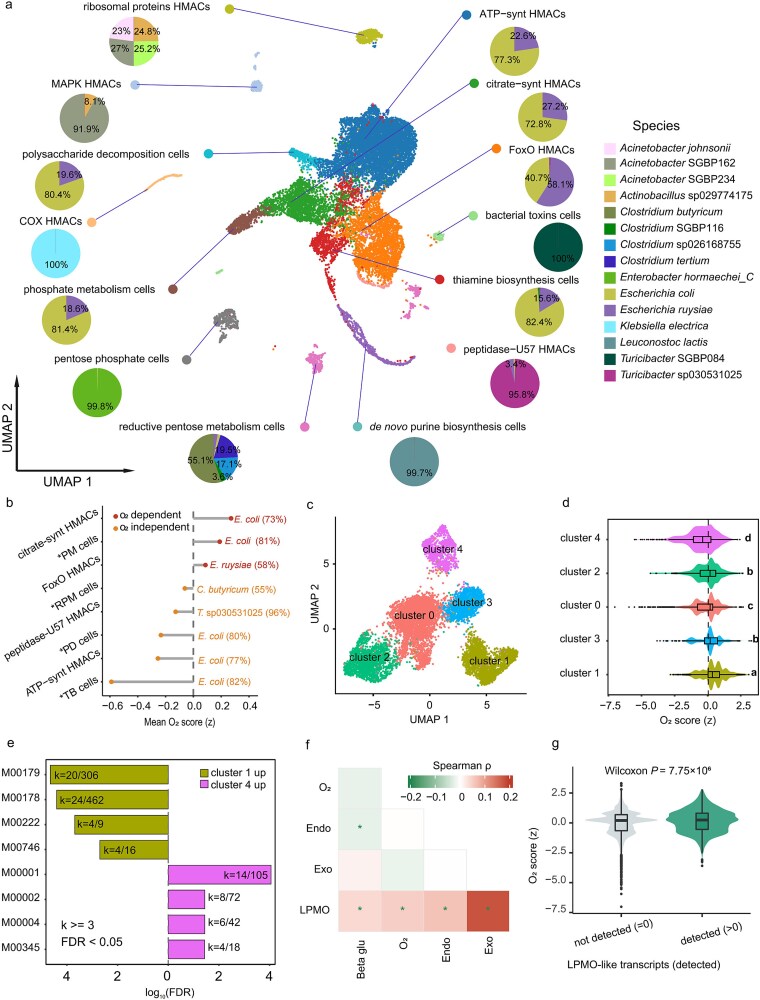
**Microbial single-cell functional clustering reveals metabolic niche partitioning in the giant panda gut;** droplet-based microbial scRNA-seq of panda fecal samples was mapped to the species-resolved Pbac v2 reference, yielding single-cell expression clusters that capture functional programs and taxonomic composition; cellulase-associated transcription is preferentially assigned to *Escherichia* spp., and *Escherichia* cells segregate into respiration-high and respiration-low transcriptional states that differentially associate with cellulase- and LPMO-like markers, consistent with the known facultative metabolism of *Escherichia coli*; (a) UMAP of all microbial cells, with cells grouped by unsupervised cluster identity; each point is one cell; for each cluster, a pie chart indicates species-level composition; HMACs, high metabolic activity cells; (b) mean oxygen score (O₂ score; z-score) per cluster for clusters observed in ≥2 samples; positive values indicate oxygen-dependent signatures (aerobic-like), negative values indicate oxygen-independent signatures (anaerobic-like); Abbreviations: PM cells, phosphate metabolism cells; RPM cells, reductive pentose metabolism cells; PD cells, polysaccharide decomposition cells; (c) UMAP of *E. coli* cells, revealing five transcriptional clusters, each present in ≥2 samples; (d) distribution of O₂ scores across the five *E. coli* clusters; letters denote groups with significant pairwise differences (distinct letters indicate *P* < .05); (e) differential gene-set enrichment contrasting *E. coli* cluster 1 versus cluster 4; only pathways with ≥3 upregulated genes and FDR <0.05 are shown; (f) pairwise Spearman correlations among O₂ score and cellulase markers: endo (endoglucanase), exo (exo-cellulase), and LPMOs (lytic polysaccharide monooxygenase); asterisks denote significant correlations (*P* < .05); (g) O₂ score comparison between cells expressing LPMOs (>0) and non-expressing cells (= 0); LPMO-positive cells exhibit higher O₂ scores (Wilcoxon rank-sum, *P* = 7.75 × 10^−6^).

To assign cellulase-associated transcripts at cellular resolution, droplet-based microbial scRNA-seq reads were mapped to the species-resolved Pbac v2 reference. The dataset comprised 16 659 cells passing QC (purity ≥50%, >15 genes per cell). Unsupervised analysis resolved 17 clusters; three with <100 cells were excluded, yielding 14 clusters (16 478 cells; mean 124.75 genes per cell) annotated by marker genes and Kyoto Encyclopedia of Genes and Genomes (KEGG) module enrichment as ribosomal proteins high metabolic activity cells (HMACs), mitogen-activated protein kinase (MAPK) HMACs, polysaccharide decomposition cells, cytochrome c oxidase (COX) HMACs, phosphate metabolism cells, pentose phosphate cells, reductive pentose metabolism cells, de novo purine biosynthesis cells, peptidase-U57 HMACs, thiamine biosynthesis cells, bacterial toxins cells, forkhead box O (FoxO) HMACs, citrate-synt HMACs, and ATP-synt HMACs (Fig. 3a). Six clusters were predominantly composed of *E. coli* or *Escherichia ruysiae*, including the largest groups (ATP-synt HMACs, citrate-synt HMACs, FoxO HMACs, and thiamine biosynthesis cells) (Supplementary Fig. S5a), indicating transcriptional heterogeneity within *Escherichia*.

At the gene-family level, *E. ruysiae* and *E. coli* together accounted for most assigned cellulase-related transcripts: GH1, 77.6%; GH3, 90.9%; GH5, 22.3%; GH9, 92.2% (Supplementary Fig. S6a, Supplementary Table S11). To validate GH5/GH9 annotations, we constructed phylogenetic trees including experimentally characterized GH5 and GH9 cellulases and our *E. coli* candidates; the candidates clustered within cellulase-containing clades (Supplementary Tables S12 and S13, Supplementary Figs S8 and S9). Additional contributions were detected from *Clostridium butyricum* and *Leuconostoc lactis*. At the cluster level, cellulase-associated expression was higher in *Escherichia*-occupied clusters, including ATP-synt HMACs, citrate-synt HMACs, and FoxO HMACs (Supplementary Fig. S6b).

To characterize cellular energy programs, a respiration-associated program score (“O₂ score”) was computed per cell from predefined gene sets and summarized for clusters present in at least two samples (*n* = 8). *Escherichia*-dominated clusters spanned the observed range, with higher scores in citrate-synt HMACs and phosphate metabolism cells and lower scores in ATP-synt HMACs and thiamine biosynthesis cells; *C. butyricum* showed low scores consistent with strict anaerobic physiology (Fig. 3b). Reclustering *E. coli* cells identified five clusters with distinct scores, with cluster 1 highest and cluster 4 lowest (Fig. 3c and d; Supplementary Fig. S6c). Differential enrichment contrasting cluster 1 versus cluster 4 indicated elevated aerobic-respiration and electron-transport modules (M00178, M00179, M00222, M00746) in the higher-score group, and higher three-carbon/fermentative pathways (M00002, M00004, M00345; glycolysis M00001) in the lower-score group (Fig. 3e). Associations between energy programs and fibrolytic features were evaluated at single-cell resolution. Across *E. coli* cells, the respiration-associated score correlated with putative LPMO-like features (Spearman, *P* < .05; Fig. 3f). Cells with detectable LPMO-like expression (>0) had higher scores than LPMOs-negative cells (Wilcoxon rank-sum, *P* = 7.75 × 10^−6^; Fig. 3g; Supplementary Fig. S6d). These data indicate that *Escherichia* subpopulations differ in respiration-linked transcriptional states and that cellulase/LPMOs markers are preferentially expressed in higher-score states, consistent with the species-resolved enterotype signature observed for ET-Ecoli (Fig. 2) and with the known facultative anaerobic lifestyle of *E. coli*.

A robustness analysis indicated that the O₂ score was not explained primarily by technical variation. Although O₂ score_z showed weak-to-moderate negative correlations with nFeature_RNA (Spearman’s ρ = −0.27, *P* = 1.1 × 10^−241^, Supplementary Fig. S7a) and nCount_RNA (ρ = −0.28, *P* = 4.5 × 10^−260^, Supplementary Fig. S7b), adjustment for these covariates largely preserved the relative ordering of cluster-level means (Supplementary Fig. S7c). Recalculation across alternative AddModuleScore settings yielded highly concordant results (Supplementary Fig. S7d), and an orthogonal UCell-based implementation showed strong agreement with the original score (ρ = 0.90, *P* < 1 × 10^−300^; Supplementary Fig. S7e and f). These analyses support interpretation of the O₂ score as a relative descriptor of respiration-associated versus anaerobic/fermentative cellular state, rather than a direct estimate of absolute oxygen concentration.

### Oxygen availability modulates cellulolytic activity of panda-derived *Escherichia in vitro*

Guided by the single-cell analysis—where *E. coli* subpopulations exhibited respiration-associated transcriptomic states and higher scores coincided with cellulase/putative LPMO-like features—we assessed oxygen effects on panda-derived isolates. Within ET-Ecoli, *E. coli* reached a median relative abundance of 38.4% in this cohort.

**Figure 4:**
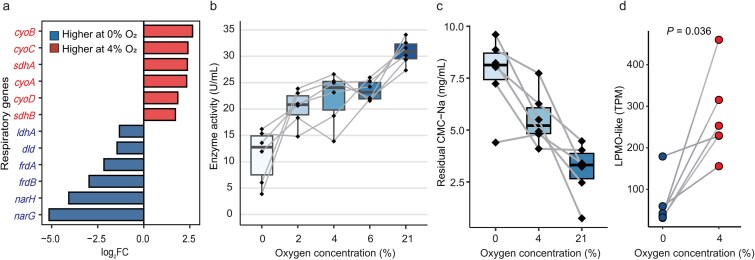
**Functional validation of oxygen-dependent cellulolytic activity in giant panda-derived *Escherichia coli* isolates;** (a) differential expression of hallmark respiration genes in *E. coli* at 0% versus 4% O₂, including aerobic-respiration genes (e.g. *cyoABCD*, *sdhAB*) and anaerobic-respiration genes (e.g. *narGH, frdAB, dld, ldhA*) (FDR <0.05; |FC| > 1); (b) fibrolytic enzyme activity measured through reducing glucose production from sodium carboxymethylcellulose (Na-CMC) hydrolysis across oxygen gradients (0%–21%); (c) residual CMC-Na quantified by a gravimetric precipitation assay across oxygen conditions; residual CMC-Na remaining after 24-h incubation of six giant panda–derived *E. coli* strains under 0%, 4%, and 21% O₂ was measured (Materials and methods); boxplots summarize strains (*n* = 6); diamonds and connecting lines indicate paired measurements per strain; paired Wilcoxon signed-rank tests: 0% versus 4% *P* = .059; 0% versus 21% *P* = .031; (d) paired plot showing LPMO-like expression under anoxic (0% O_2_) and microoxic (4% O_2_) conditions; each pair of points connected by a line corresponds to the same *E. coli* strain; expression values were normalized as transcripts per million (TPM); statistical significance was assessed using a Wilcoxon signed-rank test.

Transcriptome profiling across controlled atmospheres supported oxygen-responsive regulation of loci linked to respiration and cellulose processing. RNA-seq at 0% versus 4% O₂ showed higher expression of aerobic-respiration genes (e.g. *cyoABCD*, *sdhAB*) at 4% O₂, whereas genes associated with anaerobic respiration (e.g. *narGH*, *frdAB*, *dld*, *ldhA*) were elevated at 0% O₂ (Fig. 4a), consistent with the respiration gene sets used for program scoring.

Phenotypic assays yielded concordant patterns. Congo red staining detected no cellulose degradation under anoxia (0% O₂) and detectable degradation at 21% O₂ (Supplementary Fig. S10). Across six *E. coli* strains isolated from giant panda gut, reducing-sugar release from sodium carboxymethylcellulose increased along an oxygen gradient (0%, 2%, 4%, 6%, 21% O₂), with a significant step-increase at 4% versus 0%–2% O₂ (*P* < .05, pairwise Wilcoxon signed-rank tests) and further concentration-dependent gains between 4% and 21% O₂ (Fig. 4b). Consistent with this trend, a complementary gravimetric assay quantifying residual CMC-Na in culture supernatants after 24 h showed lower remaining CMC-Na under higher oxygen (Fig. 4c; paired Wilcoxon signed-rank tests: 0% vs*.* 4%, *P* = .059; 0% vs*.* 21%, *P* = .031), indicating increased CMC processing at 0%–21% O₂. All *E. coli* strains isolated from giant panda gut were inactive at 0% O₂ and active at ≥4% O₂ under the assay conditions (Supplementary Fig. S11).

Consistent with the single-cell associations, LPMOs-related transcripts were higher at 4% than at 0% O₂ across tested *E. coli* strains (Fig. 4d). In paired comparisons, LPMO-like expression increased at 4% O₂ (Wilcoxon signed-rank test, *P* < .05).

Under microoxic conditions (4% O₂), panda-derived *Escherichia* thus showed increased expression of cellulase-associated loci alongside measurable cellulose degradation. These *in vitro* measurements align with the respiration-linked states observed at single-cell resolution (Section *In situ* attribution of cellulase-associated transcripts by microbial scRNA-seq) and with the enrichment of respiration modules in ET-Ecoli (Section Functional specialization and core metabolism across enterotypes), although they do not permit inference of *in vivo* oxygen levels.

## Discussion

The giant panda gut presents a long-standing puzzle: a carnivore-like digestive tract supports a bamboo diet, yet canonical cellulolytic taxa are uncommon [6, 10]. We addressed this setting by establishing a high-quality, species-resolved reference (Pbac v2; 466 genomes) that improved read assignment for metagenomes and meta-transcriptomes and enabled species-level analyses. Using this reference, community profiling resolved three enterotypes with distinct carbon and energy modules; the *Escherichia*-dominated group (ET-Ecoli) was enriched for TCA and respiratory-chain components and showed higher abundance of an endo-β-1,4-glucanase marker. Microbial single-cell transcriptomics then assigned a substantial fraction of cellulase-associated transcripts *in situ* to *Escherichia* and revealed transcriptional heterogeneity within this genus, including states marked by respiration-linked programs. Tests with panda-derived isolates under defined atmospheres showed oxygen-level–dependent differences in cellulolytic readouts. In light of prior reports of limited classic fibrolytics in the panda gastrointestinal tract [5, 10], these data are consistent with abundant generalists—exemplified by *Escherichia*—contributing to fiber degradation through strain-level capacities and state-dependent expression; *in vivo* oxygen levels were not measured here.

Our resource expands cultivation and genome quality beyond recent isolate collections (e.g. 60 strains/35 species/12 genera in [41]) by sampling both feces and luminal contents, capturing taxa that are under-represented in fecal microbiota. Long-read metagenomics [4, 17, 42–44], validated for near-isolate MAG accuracy in multiple settings, yielded high-contiguity, high-completeness assemblies that, together with isolates, improved read mapping for both metagenomes and meta-transcriptomes relative to Pbac v1 [45] and pandaGUT [46]. Beyond data aggregation, this methodological pairing (isolate genomes + HiFi-MAGs) enables functional testing on living strains and supports species-level attribution, which is a recurring challenge in wildlife microbiome research.

At the community level, species-resolved enterotyping distinguished by ET-Ecoli, ET-Clos, and ET-StreA. ET-Ecoli was enriched for TCA and respiratory-chain modules (M00009, M00011, M00144, M00117) and showed higher endo-β-1,4-glucanase (EC 3.2.1.4) than other groups (Fig. 2). ET-Clos was enriched for glycolysis and reductive TCA-associated modules (M00001, M00002, M00173, M00620), and ET-StreA showed relatively higher amino- and fatty-acid biosynthesis modules. These patterns indicate metabolic partitioning in carbon and energy pathways, with ET-Ecoli and ET-Clos accounting for a larger share of the cellulase-associated potential in this cohort.

Single-cell profiling assigned most cellulase-associated transcripts *in situ* to *Escherichia*—principally *E. coli* and *E. ruysiae*—across glycoside-hydrolase families GH1, GH3, GH5, and GH9, and it revealed marked within-genus heterogeneity (Supplementary Fig. S6a). When *Escherichia* cells were examined at higher resolution, two recurrent transcriptional configurations were evident. One configuration was enriched for aerobic-respiration and electron-transport features (KEGG modules M00178, M00179, M00222, M00746), and the other was enriched for anaerobic/three-carbon pathways (M00002, M00004, M00345). Cellulase-linked markers, including putative LPMO-like features, were concentrated in the respiration-enriched configuration. These latter assignments, however, should be interpreted cautiously. Although retained here as annotation-level features and associated transcriptionally with higher O_2_ score states, their similarity to experimentally characterized LPMOs was limited, including modest sequence identity and restricted alignable regions. Accordingly, they should not be regarded as definitive functional LPMOs in *Escherichia* without direct biochemical validation. More broadly, the single-cell patterns define transcriptional programs associated with *Escherichia* subpopulations, but they do not constitute direct measurements of oxygen levels in the panda gut. Guided by these associations, panda-derived *E. coli* isolates were examined under controlled atmospheres. Across six strains (Supplementary Table S14), cellulose degradation was not detected at 0% O₂ and appeared at ≥4% O₂ with concentration-dependent increases to 21% O₂; RNA-seq contrasts showed higher expression of aerobic-respiration genes (e.g. *cyoABCD*, *sdhAB*) at 4% O₂ and higher expression of anaerobic-respiration genes (e.g. *narGH*, *frdAB*, *dld*, *ldhA*) at 0% O₂; putative LPMOs-like transcripts were also higher at 4% O₂. This oxygen-associated pattern should not be interpreted as an intrinsic biochemical requirement for glycoside-hydrolase–mediated cellulose hydrolysis. Rather, it is more consistent with oxygen-dependent physiological regulation in *E. coli*, a facultative anaerobe that transitions between anaerobic and respiratory programs according to terminal electron acceptor availability [47, 48]. We therefore interpret oxygen primarily as a modulator of cellular state that is associated with the observed cellulose-processing readouts, rather than as a direct co-substrate requirement of the hydrolytic reaction. These *in vitro* observations agree with the single-cell associations and with the respiratory-module enrichment of ET-Ecoli, but they do not establish oxygen levels *in vivo*. They are nevertheless compatible with the proposed anatomical and ecological setting of the panda gut, which may permit transient microoxic niches for facultative bacteria [49].

Viewed in a broader comparative context, our findings also connect with previous reports of cellulolytic *E. coli* from bovine and sheep rumen, where extracellular cellulase activity and rumen-adaptive physiological traits were described [50–52]. These studies suggest that cellulose-associated activity in *E. coli* is best regarded as a strain-specific and condition-dependent property of a facultative anaerobe, rather than a defining feature of a specialist fibrolytic lineage. The host setting in giant pandas is, however, distinct: they retain a carnivore-like gastrointestinal architecture with relatively rapid digesta passage, and their gut microbiota appears comparatively depleted in canonical fibrolytic taxa [6, 9, 53]. Moreover, the rumen studies, like the present work, were designed to characterize isolate physiology and niche adaptation rather than to quantify host-level energetic return attributable to *E. coli* [50, 51]. We therefore interpret panda-associated strains conservatively as contributors to cellulose processing under particular physiological states, without extending this inference to a quantitatively important role in host energy harvest.

Methodological considerations inform interpretation. Species-level expression estimation from bulk MT data was not pursued due to ortholog ambiguity and compositional limitations inherent to meta-transcriptomic profiling. The oxygen gradients used *in vitro* serve as proxies; they do not establish gut oxygen conditions. Droplet-based microbial scRNA-seq showed taxon-specific capture bias: although most of the top metagenomic taxa were represented, *S. alactolyticus*, dominant by metagenomics and 16S rRNA gene data (Supplementary Fig. S5b–d), did not pass clustering thresholds, consistent with lysis challenges in Gram-positive taxa [54]. Nonetheless, the approach captured the majority of dominant taxa: 7 of the top 10 metagenomic species were represented in the single-cell dataset. Differences in strain labels between metagenomes (e.g. *E. coli* C in the Genome Taxonomy Database (GTDB)) and single-cell assignments likely reflect analytic resolution. In this study, the attribution of fiber-associated roles is based on species-level presence and expression of relevant genes (community and single-cell data) together with oxygen-dependent cellulolytic activity observed in isolates. Our data support the involvement of *Escherichia* in cellulose processing, but do not allow inference regarding the extent to which this activity contributes to host energy harvest. We did not measure *in vivo* short-chain fatty acid (SCFA) production, carbon flux, host uptake, or animal energy balance. Therefore, the present study addresses microbial participation in cellulose processing rather than demonstrating a quantitatively significant contribution to host energy acquisition. This distinction is particularly important in giant pandas, whose overall cellulose digestibility remains limited.

Microbial scRNA-seq attributed a large fraction of cellulase-associated transcripts *in situ* to *Escherichia* and showed that these transcripts were concentrated in clusters predominantly occupied by *Escherichia* (ATP-synt, Citrate-synt, FoxO; Supplementary Fig. S4a and b). These clusters were annotated by marker genes and KEGG modules linked to energy metabolism, and they overlap with the groups in which *Escherichia* cells displayed higher respiration-program scores (Fig. 3). In this way, the single-cell data connects species identity with transcriptional programs and with cellulase/putative LPMO-like features at cellular resolution. The pattern is consistent with the ET-Ecoli signature observed at the community level (enrichment of TCA and respiratory-chain modules) and with isolate phenotypes measured under defined atmospheres, where oxygen availability modulated cellulolytic readouts (Figs 2–4). The convergence across community profiling, single-cell attribution, and strain assays strengthens the interpretation that *Escherichia* populations in this cohort harbor the capacity and the transcriptional states associated with fiber processing, without invoking unmeasured *in vivo* oxygen levels.

The integrated workflow—culturomics and HiFi metagenomes to build a species-resolved reference and isolate set, targeted phenotyping and multi-omics to test predicted activities, and microbial single-cell transcriptomics to assign activity *in situ*—provides a practical approach from community-level associations to species-level attribution in wildlife microbiomes. In a gut characterized by limited canonical fibrolytics [11, 46], generalists exemplified by *Escherichia* exhibit strain-level capacities and state-dependent expression linked to fiber processing in this dataset. Protocol refinements to improve capture of Gram-positive taxa (e.g. enzymatic pretreatment or fixation gradients [55]) and pangenome-aware strain tracking should enhance concordance between bulk and single-cell views and enable longitudinal analyses at strain resolution.

## Conclusion

We investigated fiber processing in a gut with few canonical cellulolytics by building a species-resolved reference for the giant panda microbiome (Pbac v2; 466 genomes) and integrating community, single-cell, and isolate data. Species-level profiling resolved three enterotypes (ET-Ecoli, ET-Clos, ET-StreA). Microbial single-cell transcriptomics attributed a substantial share of cellulase-associated transcripts *in situ* to *Escherichia* and revealed two configurations—respiration-linked versus three-carbon/anaerobic programs. Guided by these patterns, panda-derived *E. coli* showed oxygen-dependent cellulolytic activity *in vitro* with a threshold near 4% O₂ and concordant transcriptional shifts. These observations indicate that, in this cohort, abundant generalists can contribute to cellulose processing under microoxic conditions.

Interpretation is limited by the absence of *in vivo* oxygen or spatial measurements, the single-cell sample size, and reliance on MG/MT pairs; the nutritional impact to the host cannot be inferred here. The workflow—culturomics plus PacBio HiFi metagenomes, targeted phenotyping and multi-omics, and microbial single-cell transcriptomics—can be applied to other systems, and the open Pbac v2 resource enables reproducible re-analysis. Further work should improve capture of Gram-positive taxa and link cellular programs to *in vivo* readouts.

## Supplementary Material

Supplementary_material_wrag068

## Data Availability

Sequence data generated in this study are deposited in NCBI repositories under PRJNA1293880, PRJNA1292430, and PRJNA1402480. Short-read shotgun metagenomic and 16S rRNA gene sequencing data are available under PRJNA1293880, microbial scRNA-seq data under PRJNA1292430, and RNA-seq data from *E. coli* cultured under 0% and 4% O₂ conditions under PRJNA1402480. Assembled metagenome-assembled genomes (MAGs) and isolate genomes are permanently available through the Pbac v2 Database at https://pbac.bacbase.com. This resource provides access to both the genomic sequences and associated metadata. Complete sample metadata are also presented in the Supplementary Information.

## References

[1] Arnason U, Gullberg A, Janke A. et al. Mitogenomic analyses of caniform relationships. *Mol Phylogenet Evol* 2007;45:863–74. 10.1016/j.ympev.2007.06.019 17919938

[2] Dierenfeld E, Hintz H, Robertson J. et al. Utilization of bamboo by the giant panda. *J Nutr* 1982;112:636–41. 10.1093/jn/112.4.636 6279804

[3] Li R, Fan W, Tian G. et al. The sequence and de novo assembly of the giant panda genome. *Nature* 2010;463:311–7. 10.1038/nature08696 20010809 PMC3951497

[4] Yang S, Deng W, Li G. et al. Reference gene catalog and metagenome-assembled genomes from the gut microbiome reveal the microbial composition, antibiotic resistome, and adaptability of a lignocellulose diet in the giant panda. *Environ Res* 2024;245:118090. 10.1016/j.envres.2023.118090 38163545

[5] Zhu L, Wu Q, Dai J. et al. Evidence of cellulose metabolism by the giant panda gut microbiome. *Proc Natl Acad Sci USA* 2011;108:17714–9. 10.1073/pnas.1017956108 22006317 PMC3203778

[6] Zhang W, Liu W, Hou R. et al. Age-associated microbiome shows the giant panda lives on hemicelluloses, not on cellulose. *ISME J* 2018;12:1319–28. 10.1038/s41396-018-0051-y 29391488 PMC5931968

[7] Zhan M, Wang A, Yao Y. et al. An amateur gut microbial configuration formed in giant panda for striving to digest cellulose in bamboo: systematic evidence from intestinal digestive enzymes, functional genes and microbial structures. *Front Microbiol* 2022;13:926515. 10.3389/fmicb.2022.926515 35958139 PMC9363027

[8] Zhan M, Wang L, Xie C. et al. Succession of gut microbial structure in twin giant pandas during the dietary change stage and its role in polysaccharide metabolism. *Front Microbiol* 2020;11:551038. 10.3389/fmicb.2020.551038 33072012 PMC7537565

[9] Xue Z, Zhang W, Wang L. et al. The bamboo-eating giant panda harbors a carnivore-like gut microbiota, with excessive seasonal variations. *mBio* 2015;6:e00022–15. 10.1128/mbio.00022-15 25991678 PMC4442137

[10] Guo W, Mishra S, Zhao J. et al. Metagenomic study suggests that the gut microbiota of the giant panda (*Ailuropoda melanoleuca*) may not be specialized for fiber fermentation. *Front Microbiol* 2018;9:229. 10.3389/fmicb.2018.00229 29503636 PMC5820910

[11] Deng F, Wang C, Li D. et al. The unique gut microbiome of giant pandas involved in protein metabolism contributes to the host’s dietary adaption to bamboo. *Microbiome* 2023;11:180. 10.1186/s40168-023-01603-0 37580828 PMC10424351

[12] Ning R, Li C, Xia M. et al. Pseudomonas-associated bacteria play a key role in obtaining nutrition from bamboo for the giant panda (*Ailuropoda melanoleuca*). *Microbiol Spectrum* 2024;12:e0381923–3. 10.1128/spectrum.03819-23 PMC10913395 38305171

[13] Bolger AM, Lohse M, Usadel B. Trimmomatic: a flexible trimmer for illumina sequence data. *Bioinformatics* 2014;30:2114–20. 10.1093/bioinformatics/btu170 24695404 PMC4103590

[14] Langmead B, Salzberg SL. Fast gapped-read alignment with bowtie 2. *Nat Methods* 2012;9:357–9. 10.1038/nmeth.1923 22388286 PMC3322381

[15] Nurk S, Meleshko D, Korobeynikov A. et al. metaSpades: a new versatile metagenomic assembler. *Genome Res* 2017;27:824–34. 10.1101/gr.213959.116 28298430 PMC5411777

[16] Li H . Minimap2: pairwise alignment for nucleotide sequences. *Bioinformatics* 2018;34:3094–100. 10.1093/bioinformatics/bty191 29750242 PMC6137996

[17] Deng F, Han Y, Li M. et al. HiFi based metagenomic assembly strategy provides accuracy near isolated genome resolution in MAG assembly. *iMetaOmics* 2025;2:e70041. 10.1002/imo2.70041 41676447 PMC12806023

[18] Kang DD, Li F, Kirton E. et al. MetaBAT 2: an adaptive binning algorithm for robust and efficient genome reconstruction from metagenome assemblies. *PeerJ* 2019;7:e7359. 10.7717/peerj.7359 31388474 PMC6662567

[19] Olm MR, Brown CT, Brooks B. et al. dRep: a tool for fast and accurate genomic comparisons that enables improved genome recovery from metagenomes through de-replication. *ISME J* 2017;11:2864–8. 10.1038/ismej.2017.126 28742071 PMC5702732

[20] Chklovski A, Parks DH, Woodcroft BJ. et al. CheckM2: a rapid, scalable and accurate tool for assessing microbial genome quality using machine learning. *Nat Methods* 2023;20:1203–12. 10.1038/s41592-023-01940-w 37500759

[21] Chan PP, Lin BY, Mak AJ. et al. tRNAscan-SE 2.0: improved detection and functional classification of transfer RNA genes. *Nucleic Acids Res* 2021;49:9077–96. 10.1093/nar/gkab688 34417604 PMC8450103

[22] Chaumeil P-A, Mussig AJ, Hugenholtz P. et al. GTDB-Tk: a toolkit to classify genomes with the genome taxonomy database. *Bioinformatics* 2019;36:1925–7. 10.1093/bioinformatics/btz848 31730192 PMC7703759

[23] Asnicar F, Thomas AM, Beghini F. et al. Precise phylogenetic analysis of microbial isolates and genomes from metagenomes using PhyloPhlAn 3.0. *Nat Commun* 2020;11:2500. 10.1038/s41467-020-16366-7 32427907 PMC7237447

[24] Letunic I, Bork P. Interactive tree of life (iTOL) v5: an online tool for phylogenetic tree display and annotation. *Nucleic Acids Res* 2021;49:W293–6. 10.1093/nar/gkab301 33885785 PMC8265157

[25] Wood DE, Lu J, Langmead B. Improved metagenomic analysis with Kraken 2. *Genome Biol* 2019;20:257. 10.1186/s13059-019-1891-0 31779668 PMC6883579

[26] Lu J, Breitwieser FP, Thielen P. et al. Bracken: estimating species abundance in metagenomics data. *PeerJ Comput Sci* 2017;3:e104. 10.7717/peerj-cs.104 PMC12016282 40271438

[27] Li W, Godzik A. CD-HIT: a fast program for clustering and comparing large sets of protein or nucleotide sequences. *Bioinformatics* 2006;22:1658–9. 10.1093/bioinformatics/btl158 16731699

[28] Cantalapiedra CP, Hernández-Plaza A, Letunic I. et al. eggNOG-mapper v2: functional annotation, orthology assignments, and domain prediction at the metagenomic scale. *Mol Biol Evol* 2021;38:5825–9. 10.1093/molbev/msab293 34597405 PMC8662613

[29] Patro R, Duggal G, Love MI. et al. Salmon provides fast and bias-aware quantification of transcript expression. *Nat Methods* 2017;14:417–9. 10.1038/nmeth.4197 28263959 PMC5600148

[30] Zheng J, Ge Q, Yan Y. et al. dbCAN3: automated carbohydrate-active enzyme and substrate annotation. *Nucleic Acids Res* 2023;51:W115–21. 10.1093/nar/gkad328 37125649 PMC10320055

[31] Drula E, Garron M-L, Dogan S. et al. The carbohydrate-active enzyme database: functions and literature. *Nucleic Acids Res* 2022;50:D571–7. 10.1093/nar/gkab1045 34850161 PMC8728194

[32] Zhang YHP, Hong J, Ye X. Cellulase assays. In: Mielenz JR (ed.), Biofuels: Methods and Protocols, Totowa, NJ: Humana Press, 2009, 213–31. 10.1007/978-1-60761-214-8_14

[33] Dobin A, Davis CA, Schlesinger F. et al. STAR: ultrafast universal RNA-seq aligner. *Bioinformatics* 2013;29:15–21. 10.1093/bioinformatics/bts635 23104886 PMC3530905

[34] Butler A, Hoffman P, Smibert P. et al. Integrating single-cell transcriptomic data across different conditions, technologies, and species. *Nat Biotechnol* 2018;36:411–20. 10.1038/nbt.4096 29608179 PMC6700744

[35] Prokka ST . Rapid prokaryotic genome annotation. *Bioinformatics* 2014;30:2068–9. 10.1093/bioinformatics/btu153 24642063

[36] Page AJ, Cummins CA, Hunt M. et al. Roary: rapid large-scale prokaryote pan genome analysis. *Bioinformatics* 2015;31:3691–3. 10.1093/bioinformatics/btv421 26198102 PMC4817141

[37] Chen S, Zhou Y, Chen Y. et al. Fastp: an ultra-fast all-in-one fastq preprocessor. *Bioinformatics* 2018;34:i884–90. 10.1093/bioinformatics/bty560 30423086 PMC6129281

[38] Kim D, Paggi JM, Park C. et al. Graph-based genome alignment and genotyping with HISAT2 and hisat-genotype. *Nat Biotechnol* 2019;37:907–15. 10.1038/s41587-019-0201-4 31375807 PMC7605509

[39] Li H, Handsaker B, Wysoker A. et al. The sequence alignment/map format and SAMtools. *Bioinformatics* 2009;25:2078–9. 10.1093/bioinformatics/btp352 19505943 PMC2723002

[40] Liao Y, Smyth GK, Shi W. featureCounts: an efficient general-purpose read summarization program. *Bioinformatics* 2014;30:923–30. 10.1093/bioinformatics/btt656 24227677

[41] Zhang W, Zheng L, Xie J. et al. The giant panda gut harbors a high diversity of lactic acid bacteria revealed by a novel culturomics pipeline. *mSystems* 2024;9:e00520–4. 10.1128/msystems.00520-24 38920380 PMC11265448

[42] Kim CY, Ma J, Lee I. HiFi metagenomic sequencing enables assembly of accurate and complete genomes from human gut microbiota. *Nat Commun* 2022;13:6367. 10.1038/s41467-022-34149-0 36289209 PMC9606305

[43] Zhang Y, Jiang F, Yang B. et al. Improved microbial genomes and gene catalog of the chicken gut from metagenomic sequencing of high-fidelity long reads. *GigaScience* 2022;11:giac116. 10.1093/gigascience/giac116 36399059 PMC9673493

[44] Tao Y, Xun F, Zhao C. et al. Improved assembly of metagenome-assembled genomes and viruses in Tibetan saline lake sediment by HiFi metagenomic sequencing. *Microbiol Spectrum* 2023;11:e03328–2. 10.1128/spectrum.03328-22 PMC9927493 36475839

[45] Deng F, Han Y, Huang Y. et al. A comprehensive analysis of antibiotic resistance genes in the giant panda gut. *iMeta* 2024;3:e171. 10.1002/imt2.171 38868505 PMC10989137

[46] Huang G, Shi W, Wang L. et al. Pandagut provides new insights into bacterial diversity, function, and resistome landscapes with implications for conservation. *Microbiome* 2023;11:221. 10.1186/s40168-023-01657-0 37805557 PMC10559513

[47] Unden G, Steinmetz PA, Degreif-Dünnwald P. The aerobic and anaerobic respiratory chain of *Escherichia coli* and *Salmonella enterica*: enzymes and energetics. *EcoSal Plus* 2014;6:37. 10.1128/ecosalplus.esp-0005-2013 26442941

[48] Brown AN, Anderson MT, Bachman MA. et al. The ArcAB two-component system: function in metabolism, redox control, and infection. *Microbiol Mol Biol Rev* 2022;86:e0011021–1. 10.1128/mmbr.00110-21 35442087 PMC9199408

[49] Yao R, Yang Z, Zhang Z. et al. Are the gut microbial systems of giant pandas unstable? *Heliyon* 2019;5:e02480. 10.1016/j.heliyon.2019.e02480 31687574 PMC6819816

[50] Pang J, Liu Z-Y, Hao M. et al. An isolated cellulolytic *Escherichia coli* from bovine rumen produces ethanol and hydrogen from corn straw. *Biotechnol Biofuels* 2017;10:165. 10.1186/s13068-017-0852-7 28652866 PMC5483281

[51] Pang J, Liu Z, Zhang Q. et al. Systematic analysis of *Escherichia coli* isolates from sheep and cattle suggests adaption to the rumen niche. *Appl Environ Microbiol* 2020;86:e01417–20. 10.1128/AEM.01417-20 32801187 PMC7531959

[52] Mohammadabadi T, Harsini M, Motamedi H. et al. Processing of the lignocellulosic matters with cellulolytic bacteria isolated from the one hump camel foregut. *Iran Vet J* 2024;20:56–67. 10.22055/ivj.2023.391363.2575

[53] Scoma A, Khor WC, Coma M. et al. Substrate-dependent fermentation of bamboo in giant panda gut microbiomes: leaf primarily to ethanol and pith to lactate. *Front Microbiol* 2020;11:530. 10.3389/fmicb.2020.00530 32300339 PMC7145396

[54] Rohde M . The Gram-positive bacterial cell wall. *Microbiol Spectrum* 2019;7:21. 10.1128/microbiolspec.gpp3-0044-2018 PMC11086966 31124431

[55] Ma P, Amemiya HM, He LL. et al. Bacterial droplet-based single-cell RNA-seq reveals antibiotic-associated heterogeneous cellular states. *Cell.* 2023;186:877–891.e14. 10.1016/j.cell.2023.01.002 36708705 PMC10014032

