# Supplementary material for: Microoxic conditions promote Escherichia-associated cellulase expression in the giant panda gut
Source: ISME J. 2026 Apr 2;20(1):wrag068. doi: 10.1093/ismejo/wrag068 (PMC13099265; doi:10.1093/ismejo/wrag068)
Supplement: Supplementary_material_wrag068 [file supplementary_material_wrag068.zip › Supplementary Note.docx]

**Supplementary Note 1. Validation of the enterotype clustering**

To evaluate the stability and generalizability of the enterotype classification, we assessed clustering performance across a range of cluster numbers (k = 2–6). Species-level relative abundance data were Hellinger-transformed, and two complementary indices were applied the Calinski–Harabasz (CH) index (based on k-means clustering in Euclidean space) and the mean silhouette width (based on PAM clustering with Bray–Curtis distances). Across k values, the CH index peaked at k = 3 (CH = 61.4), while the mean silhouette width was slightly higher at k = 2 (0.352) but remained comparably high at k = 3 (0.332; Δ = 0.020, ~5.7%). Considering the stronger CH support, the minor decline in silhouette, and the biological interpretability of the resulting groups, a three-cluster solution was selected. Bootstrap validation (B = 500) further confirmed the robustness of this solution, with per-cluster Jaccard similarities of 0.896, 0.876, and 0.910, all above the commonly accepted stability threshold of 0.75 (Supplementary Figure S3). These results demonstrate that the three-enterotype structure is both statistically stable and biologically meaningful.

**Supplementary Note 2 — Robustness Analysis of Cellulase-Associated Signals**

To assess the robustness of cellulase-associated enzyme signals to enterotype definitions, we re-clustered the 142 samples under two compositional frameworks: (A) Bray–Curtis distance after Hellinger transformation and (B) Euclidean distance under centered log-ratio (CLR) transformation (Aitchison geometry). Partitioning around medoids (PAM, k = 3) was applied in both cases, and the cluster corresponding to the “CellulaseHigh” enterotype was identified based on the highest mean abundance of EC 3.2.1.4 (endo-1,4-β-glucanase). For each framework, we compared cellulase-related ECs (3.2.1.4) between the CellulaseHigh and Other enterotypes using Wilcoxon rank-sum tests (one-sided, CellulaseHigh > Others). False discovery rate (FDR) was controlled within each compositional definition using the Benjamini–Hochberg procedure. Effect sizes were expressed as median differences in relative abundance. Analyses were implemented in R (version 4.3) using the vegan.

EC 3.2.1.4 remained significantly enriched in the CellulaseHigh enterotype across both compositional definitions (Bray–Hellinger: *p* = 0.014, q = 0.04; Aitchison/CLR: *p* = 0.0136, q = 0.04), with consistent positive effect sizes (+1876–2588). These results indicate that the enrichment of EC 3.2.1.4 is robust to the choice of compositional transformation and distance metric, supporting its biological relevance and reproducibility across enterotype definitions.

**Supplementary Figures and Tables:**

**Figure S1. Culturomics workflow for giant panda gut microbiota isolation and characterization.**

**Figure S2. Pbac v2 outperforms existing references in genome quality and mapping efficiency.** (a) Comparative genome quality metrics showing that Pbac v2 (augmented with isolate genomes and PacBio HiFi MAGs) has a higher fraction of high-quality genomes than existing reference datasets; improvements include both newly recovered species and higher-quality representatives of previously known species. (b) Mapping efficiency benchmarking using metagenomic (MG) and meta-transcriptomic (MT) data (*n* = 14 samples). Bar plot depicts alignment rates (%) when filtering genomes by completeness (x-axis) and contamination thresholds.

**Figure S3. Validation of enterotype clustering and selection of the optimal cluster number.** (a) Calinski–Harabasz (CH) index computed from k-means clustering in Euclidean space using Hellinger-transformed species-level relative abundance profiles across 142 metagenomic samples, evaluated for k = 2–6. The CH index peaks at k = 3 (CH = 61.4). (b) Mean silhouette width calculated from PAM clustering with Bray–Curtis dissimilarity across the same k range. The silhouette is highest at k = 2 (0.352) but remains comparably high at k = 3 (0.332). The vertical dashed line indicates the selected three-cluster (three-enterotype) solution; filled circles denote the peak value of each metric.

**Figure S4. Phylogenomic tree of *Clostridium* SGBP116 assembled via three independent sequencing strategies alongside reference genomes from the *Clostridium* genus.** The phylogeny was inferred using GTDB-Tk v2.4.1 with subsequent visualization in Interactive Tree of Life (iTOL, v6.5.2).

**Figure S5. Species-level taxonomic profiling using different sequencing technologies.** (a) Log10-transformed species abundance across cell clusters. X-axis: 14 transcriptionally distinct clusters; Y-axis: top 15 species ranked by cross-cluster prevalence. (b) Metagenomic species composition (top 15 taxa) across four giant panda fecal samples. Stacked bars show relative abundance at the species level, with samples grouped by individual host. (c) Species composition of four giant panda fecal samples derived from full-length 16S rRNA gene sequencing (PacBio CCS, circular consensus sequencing). Stacked bars show relative abundances of top 15 species. (d) Analogous profile generated by V3-V4 hypervariable region sequencing (NovaSeq 6000 System, 2 × 300 bp; Deblur2 denoising).

**Figure S6. Single-cell analyses supporting oxygen-linked cellulase programs in the giant panda gut.** (a) Relative transcript contributions of dominant species to cellulolytic glycoside hydrolase families (GH1, GH3, GH5, GH6, GH9). (b) Cluster-level expression of key endoglucanase and β-glucosidase genes, highlighting active subpopulations dominated by *Escherichia* spp. (c) Trajectory inference on *Escherichia coli* scRNA-seq identifies four lineages spanning the transcriptional manifold, providing a framework to map cellular state transitions relevant to oxygen-responsive programs. (d) Among LPMOs-positive *E. coli* cells (UCell score > 0), the oxygen score (z-scored module signature) shows a weak positive association with the LPMOs UCell score (Spearman’s ρ = 0.06, *P* = 0.068; two-sided). Each point represents one cell. The blue regression line is overlaid to visualize the overall trend between LPMO expression and O_2_ score, and the shaded area indicates the confidence interval of the fitted line.

**Figure S7. Technical robustness of the O₂ score.** (a, b) O₂ score_z versus nFeature_RNA (a) and nCount_RNA (b). Each point represents one cell; lines indicate least-squares fits for visualization only. O₂ score_z showed weak-to-moderate negative correlations with nFeature_RNA (ρ = −0.27, *P* = 1.1 × 10⁻²⁴¹, *n* = 14,462) and nCount_RNA (ρ = −0.28, *P* = 4.5 × 10⁻²⁶⁰, *n* = 14,462). (c) Raw and covariate-adjusted cluster means of O₂ score_z. Cluster ordering was largely preserved after adjustment. (d) O₂ score_z from AddModuleScore versus UCell. The two approaches were highly concordant (ρ = 0.90, *P* < 1 × 10⁻³⁰⁰, *n* = 14,462). (e, f) Parameter sensitivity of AddModuleScore. O₂ scores recalculated across alternative nbin, ctrl, and seed settings remained highly concordant with the original score at the cell level (e; ρ = 0.81–0.97) and cluster-mean level (f; ρ = 0.76–1.00). Boxes show medians and interquartile ranges; whiskers indicate 1.5 × IQR.

**Figure S8. Phylogenetic placement of GH5 cellulase candidates predicted in *E. coli*.** Phylogenetic tree reconstructed from a multiple sequence alignment of amino-acid sequences of experimentally characterized GH5 cellulases (CAZyme database) together with the GH5 candidate proteins selected for prediction in *E. coli* in this study. *E. coli* candidates are highlighted, and clades containing characterized cellulases are indicated.

**Figure S9. Phylogenetic placement of GH9 cellulase candidates predicted in *E. coli*.** Phylogenetic tree inferred from amino-acid sequences of experimentally characterized GH9 cellulases (CAZyme database) together with the GH9 candidate enzymes selected for prediction in *E. coli*. The *E. coli* candidates are highlighted.

**Figure S10.** Cellulose degradation capacity assessed by Congo red assay for *E. coli* cultured at 0% versus 21% O₂.

**Figure S11.** Cellulose degradation capacity assessed by Congo red assay for six *E. coli* strains cultured at 0% versus 4% O₂.

**Table S1.** Overview of post-processing sequencing data from PacBio HiFi platforms

**Table S2.** Overview of samples for culturomics

**Table S3.** List of culture medium and incubation conditions used in this study

**Table S4.** Summary of giant panda fecal metagenomic datasets included in this study

**Table S5.** Genomic characteristics and taxonomic classification of all isolated bacterial strains

**Table S6.** Representative MAGs derived from PacBio HiFi sequencing with 99% cluster identity

**Table S7.** Species representatives (95% identity clusters) in Pbac v2 giant panda gut microbiome reference database

**Table S8.** Metagenome-derived species relative abundance matrix

**Table S9.** Sample × gene relative abundance matrix derived from metagenomes (rows: genes, columns: samples)

**Table S10.** Differentially enriched KEGG modules among enterotypes identified by LEfSe analysis

**Table S11.** CAZyme annotation and degradation potential matrix of giant panda gut microbial genomes

**Table S12.** Accession numbers of protein sequences used for phylogenetic analysis of *E. coli* GH5 proteins

**Table S13.** Accession numbers of protein sequences used for phylogenetic analysis of *E. coli* GH9 proteins

**Table S14.** Genome quality and GH5/GH9 and putative LPMO-like gene counts in six panda-derived *E. coli* isolates
