# Supplementary figures and images for: Microoxic conditions promote Escherichia-associated cellulase expression in the giant panda gut
Source: ISME J. 2026 Apr 2;20(1):wrag068. doi: 10.1093/ismejo/wrag068 (PMC13099265; doi:10.1093/ismejo/wrag068)

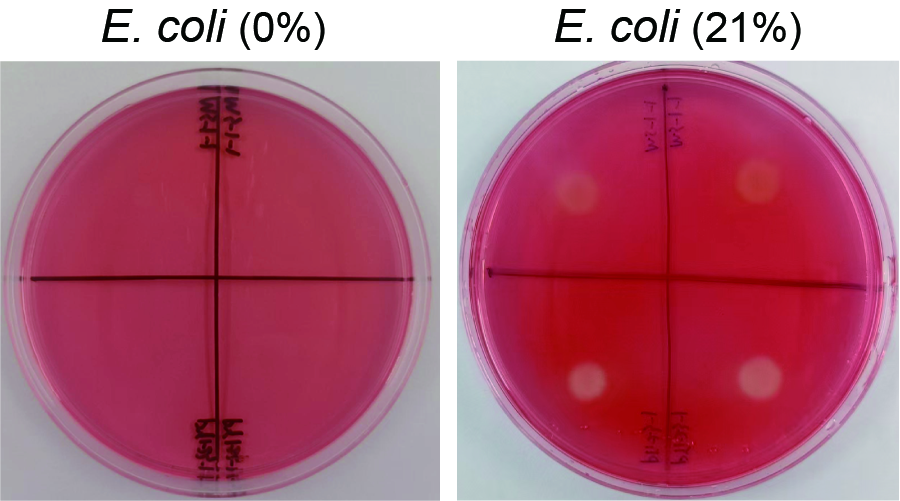

Supplement: Supplementary_material_wrag068 [file supplementary_material_wrag068.zip › Figure S10 - R3.tif]

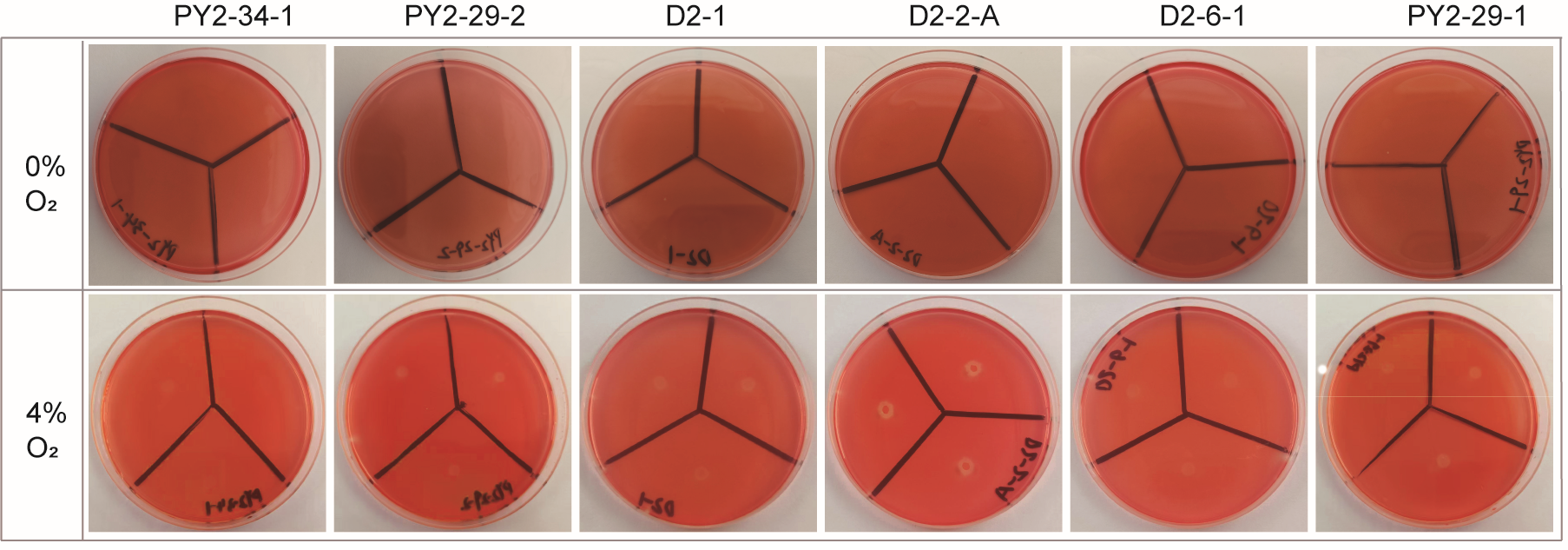

Supplement: Supplementary_material_wrag068 [file supplementary_material_wrag068.zip › Figure S11.tif]

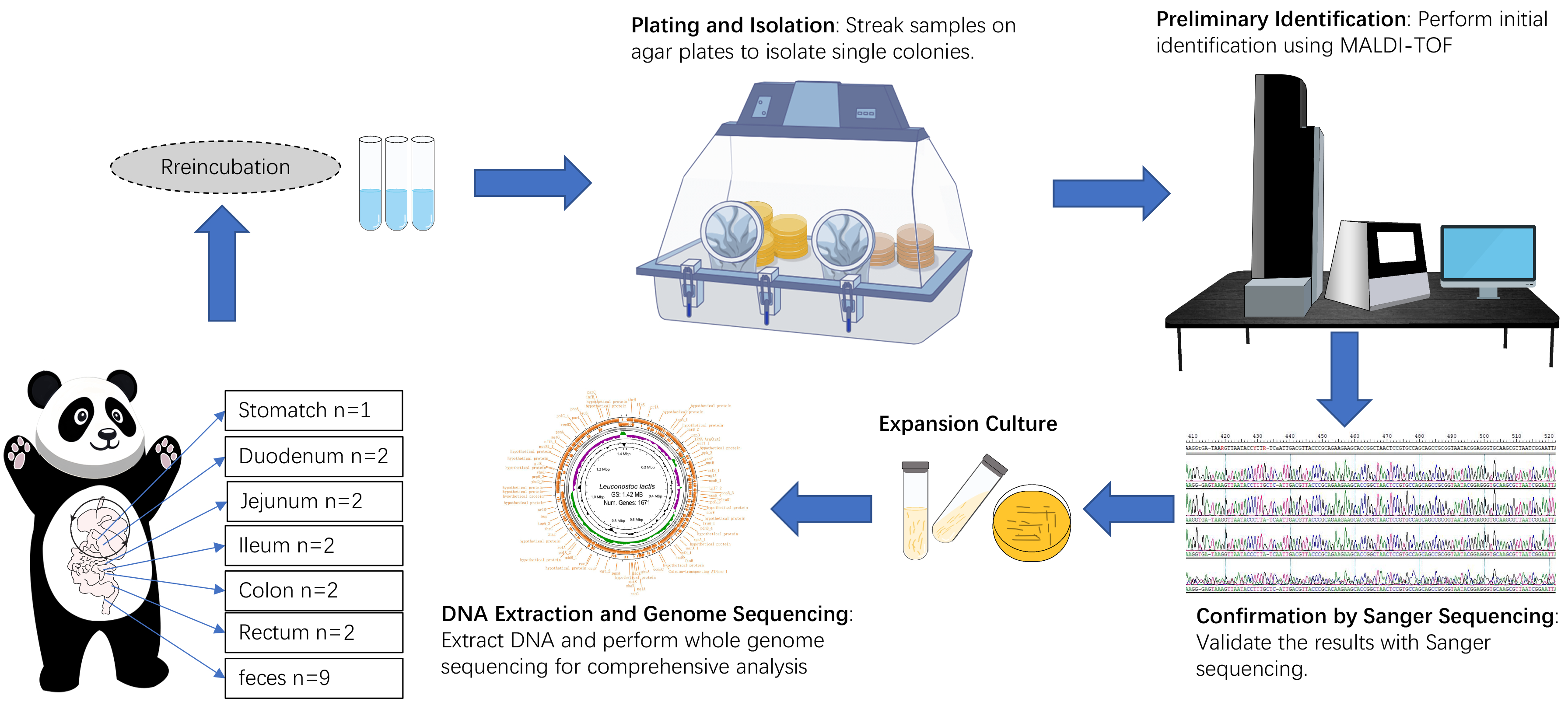

Supplement: Supplementary_material_wrag068 [file supplementary_material_wrag068.zip › Figure S1.tif]

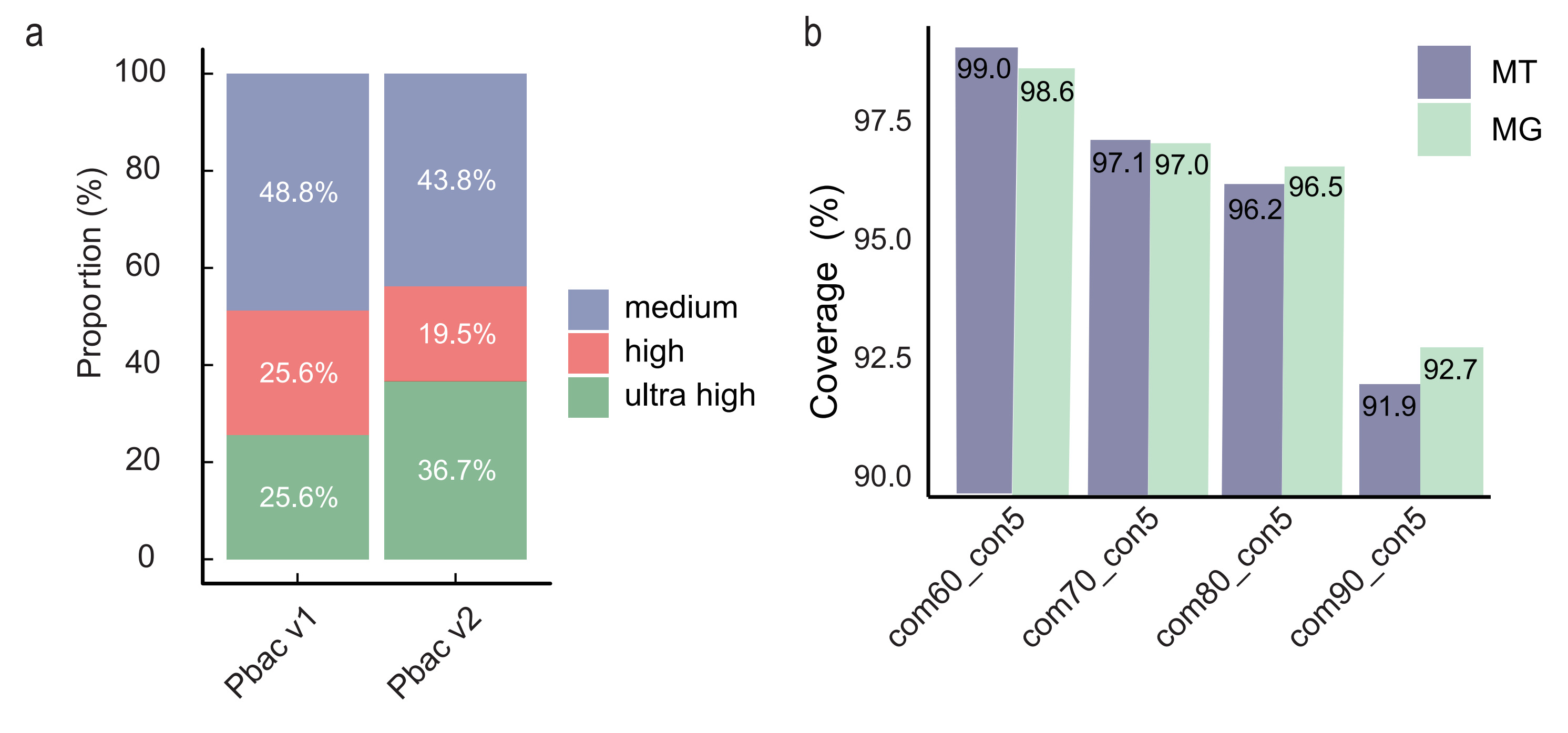

Supplement: Supplementary_material_wrag068 [file supplementary_material_wrag068.zip › Figure S2. R4.tif]

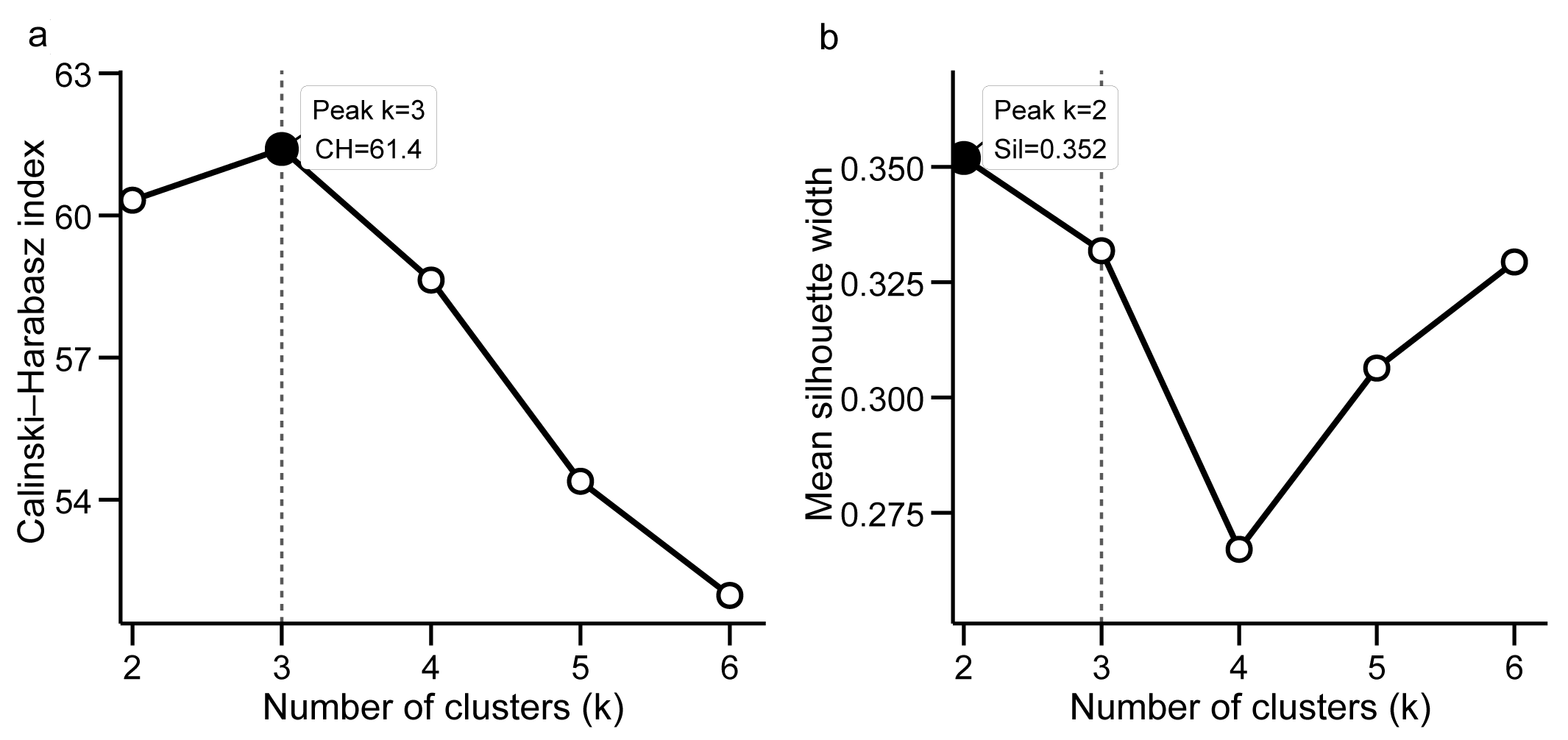

Supplement: Supplementary_material_wrag068 [file supplementary_material_wrag068.zip › Figure S3. R4.tif]

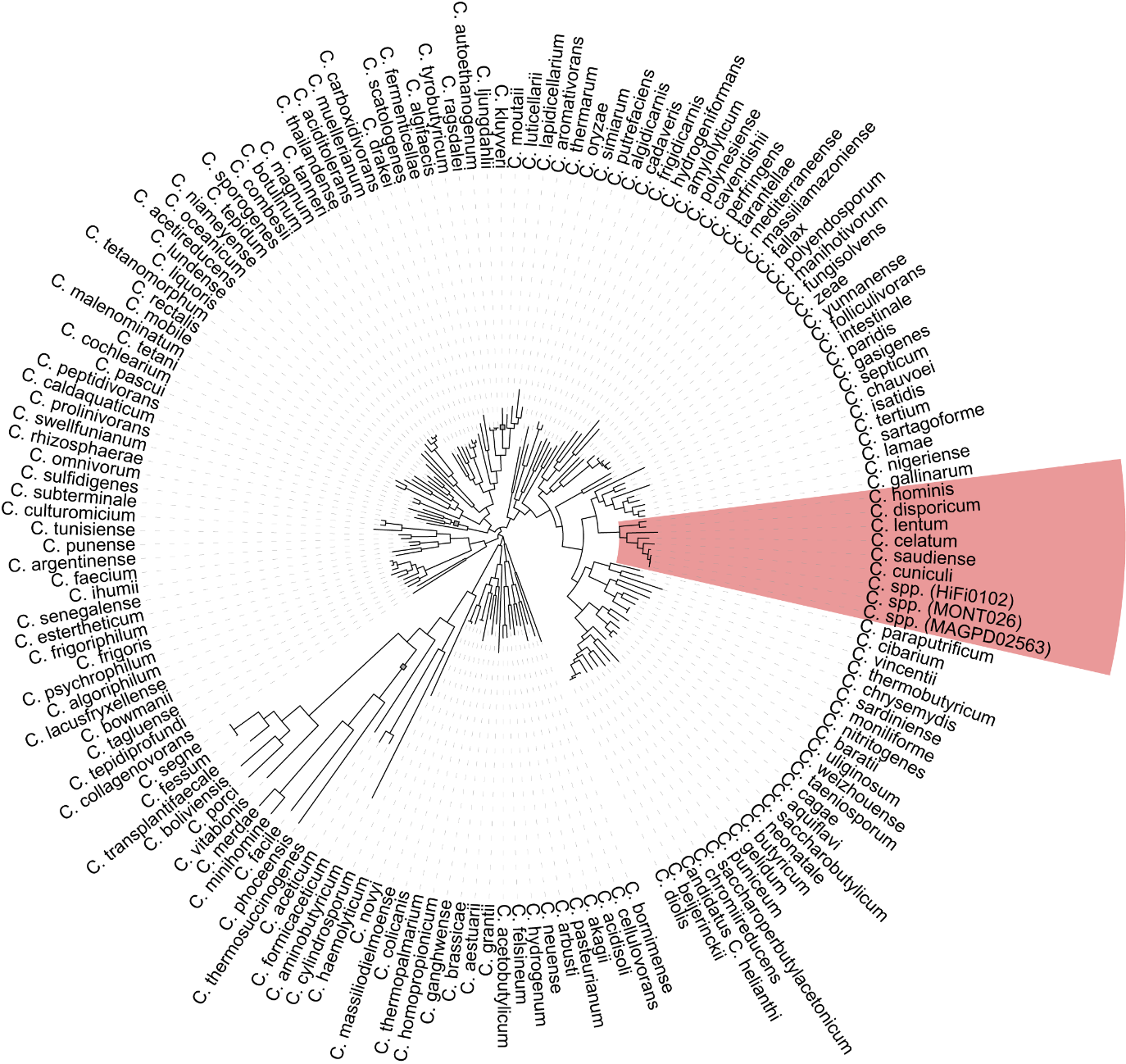

Supplement: Supplementary_material_wrag068 [file supplementary_material_wrag068.zip › Figure S4.tif]

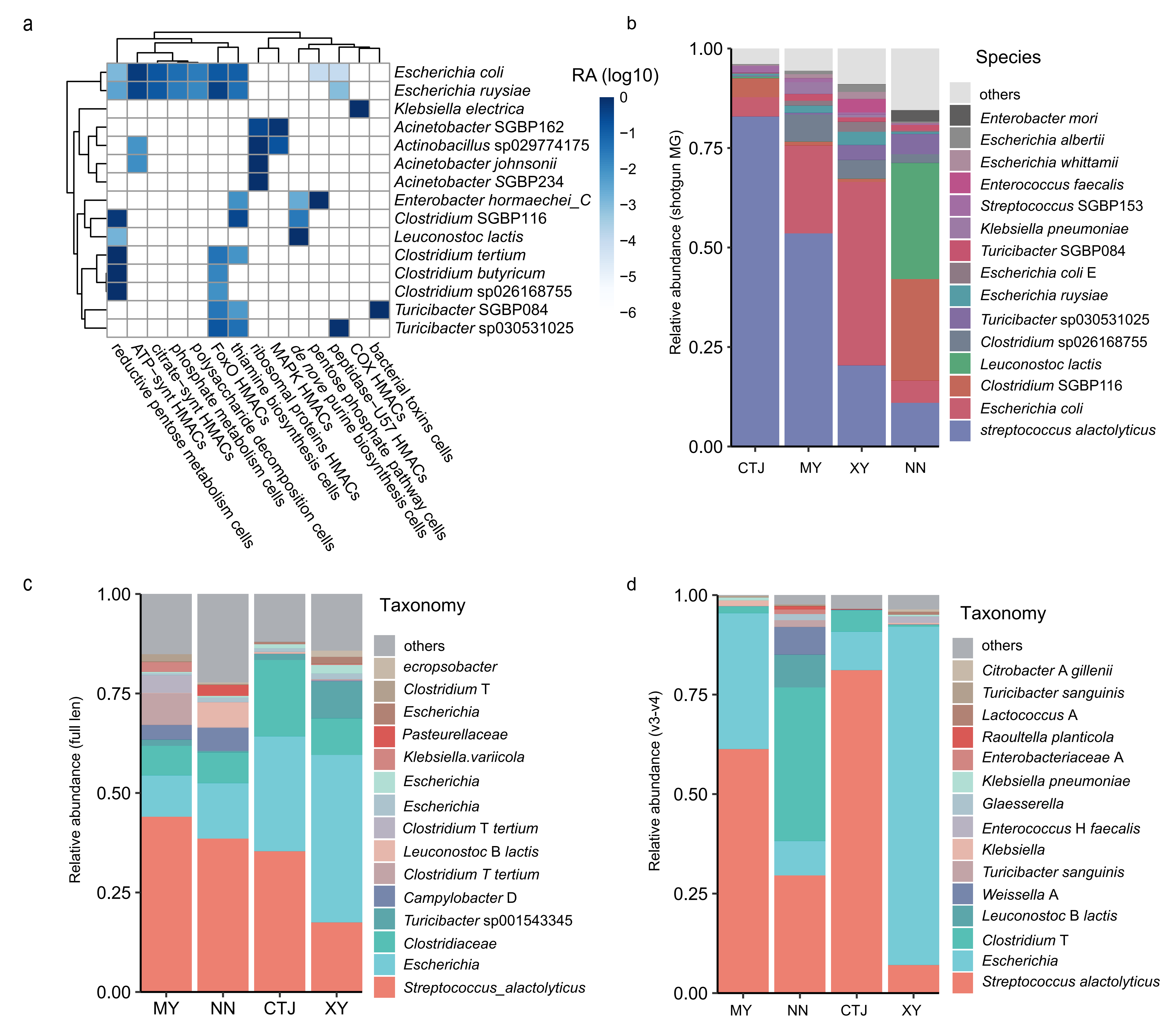

Supplement: Supplementary_material_wrag068 [file supplementary_material_wrag068.zip › Figure S5. R4.tif]

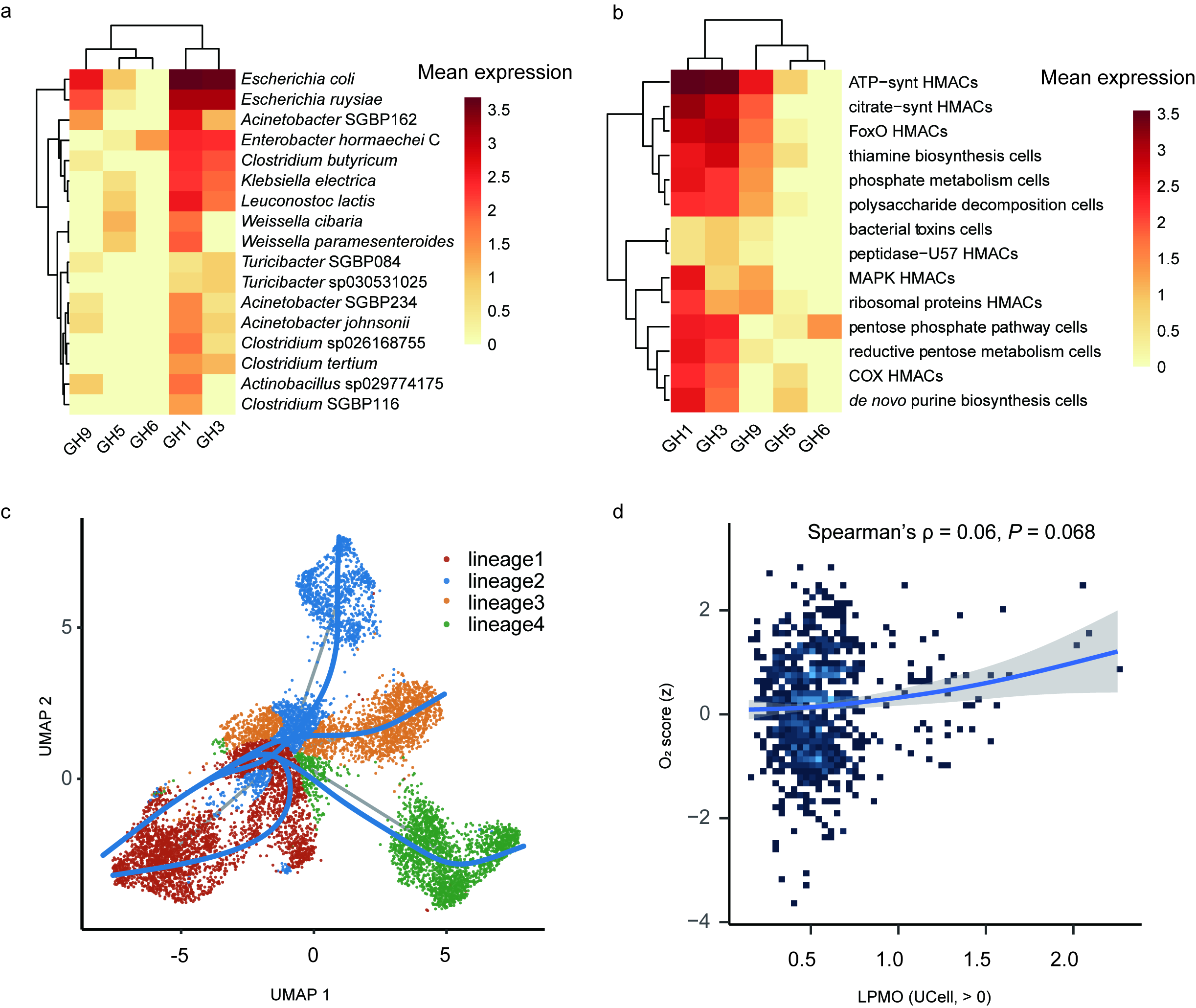

Supplement: Supplementary_material_wrag068 [file supplementary_material_wrag068.zip › Figure S6 - R4.tif]

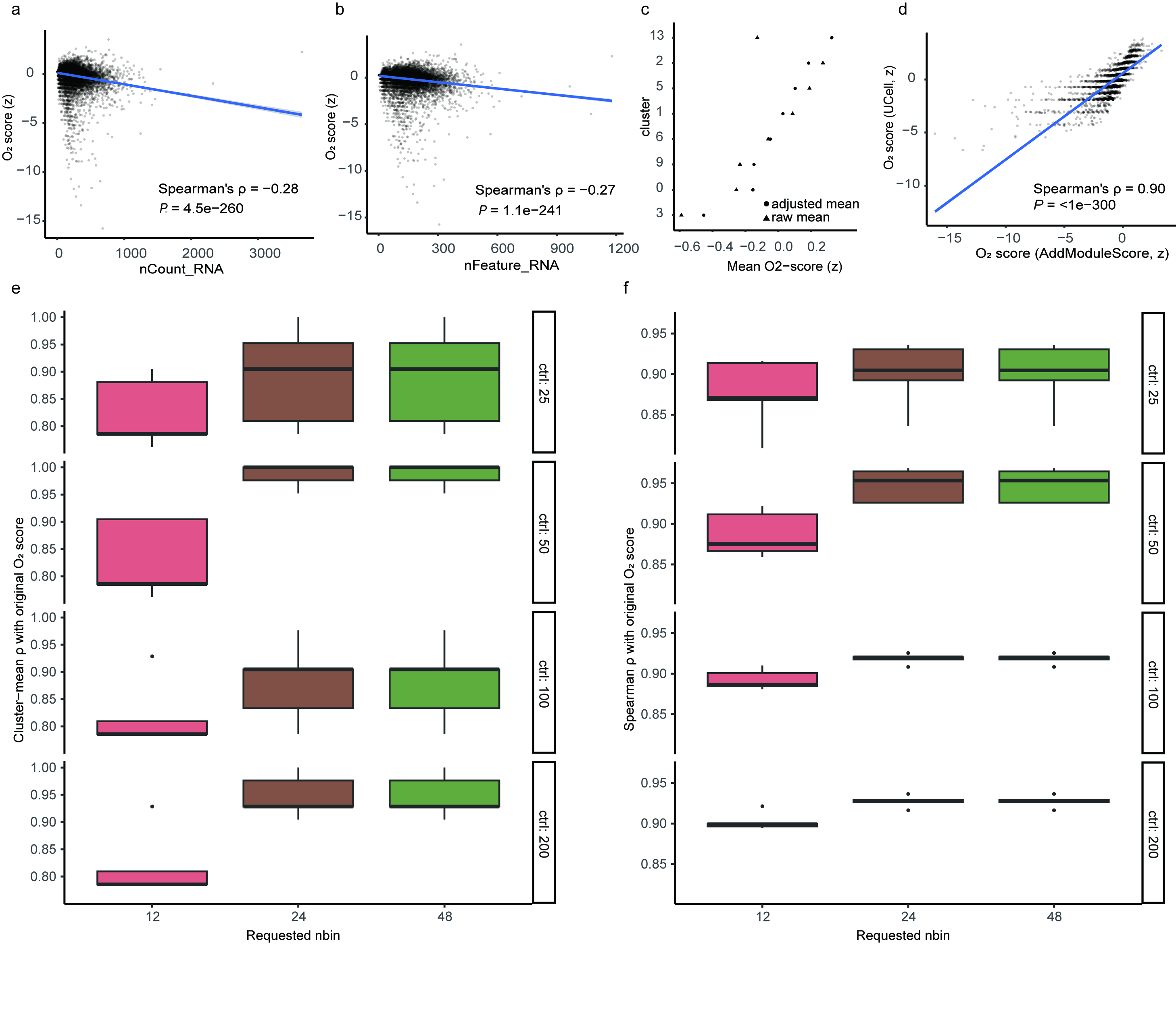

Supplement: Supplementary_material_wrag068 [file supplementary_material_wrag068.zip › Figure S7. R4.tif]

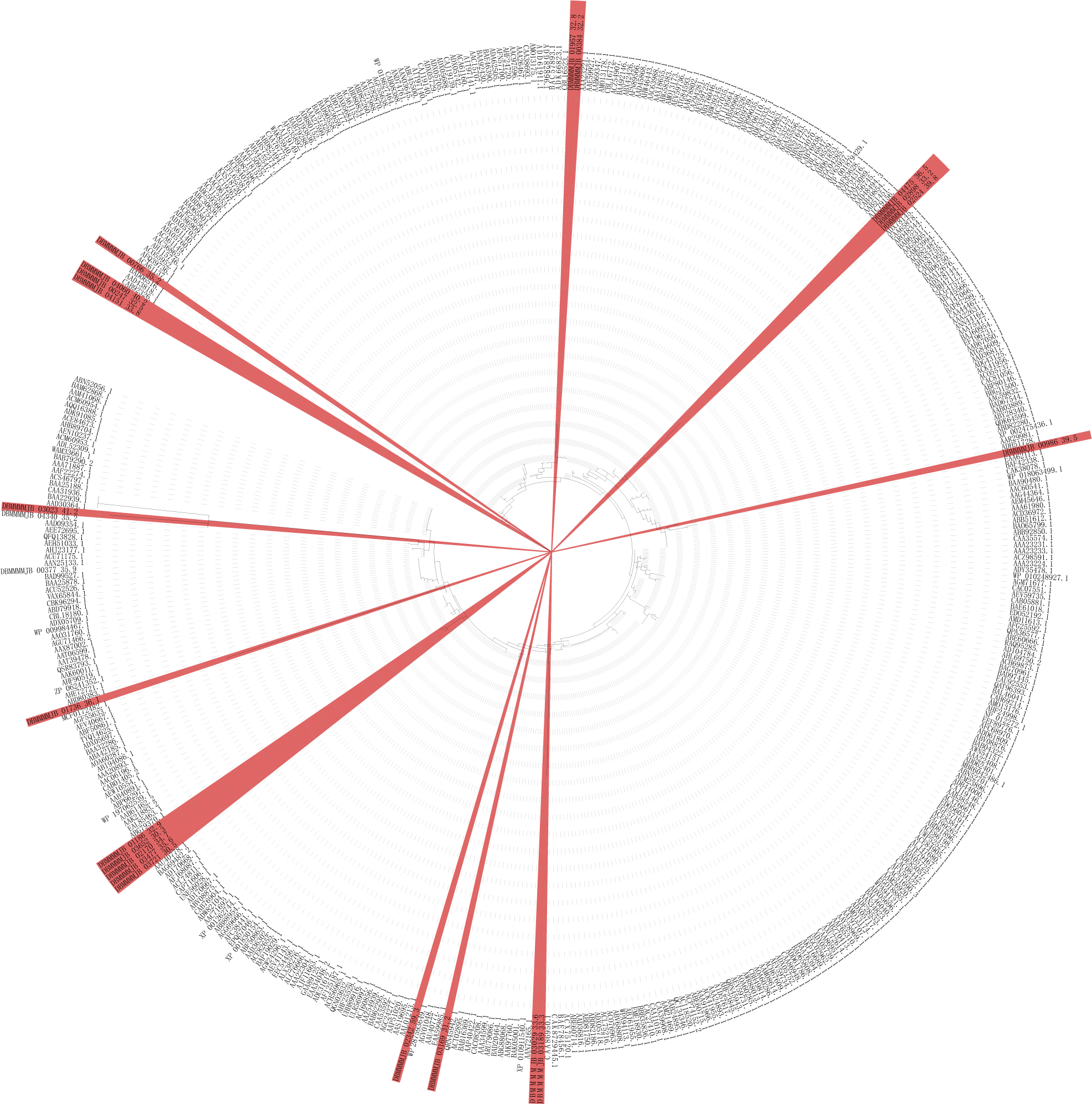

Supplement: Supplementary_material_wrag068 [file supplementary_material_wrag068.zip › Figure S8.tif]

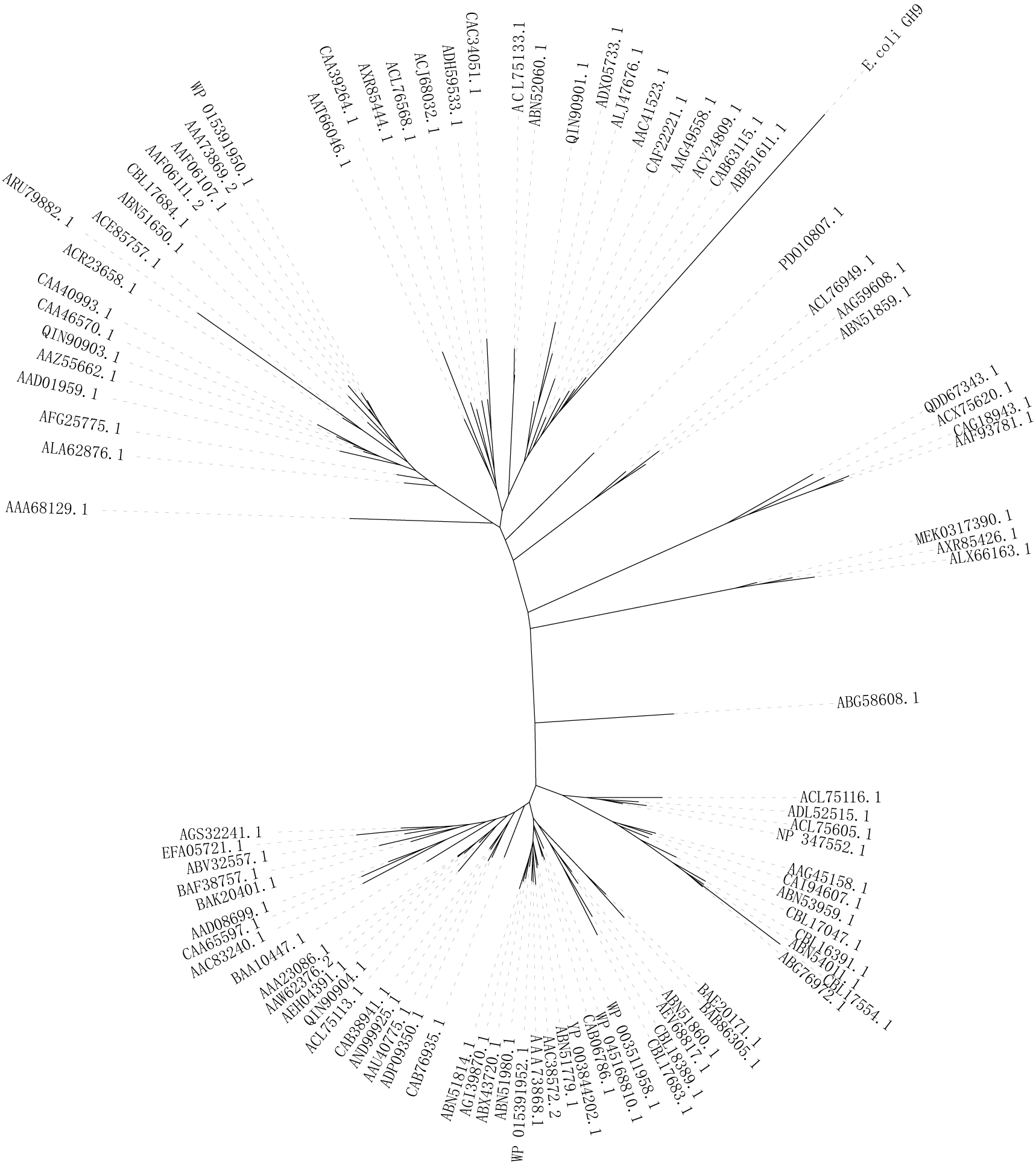

Supplement: Supplementary_material_wrag068 [file supplementary_material_wrag068.zip › Figure S9.tif]
